# Tailoring the Anisotropic Oxygen Transport Properties in Bulk Ceramic Membranes Based on a Ruddlesden–Popper Oxide by Applying Magnetic Fields

**DOI:** 10.1002/advs.202411251

**Published:** 2025-02-03

**Authors:** Giamper Escobar Cano, Motohide Matsuda, Zhijun Zhao, Frank Steinbach, Bernd Breidenstein, Hilke Petersen, Andreas Graff, Marc Widenmeyer, Anke Weidenkaff, Armin Feldhoff

**Affiliations:** ^1^ Institute of Physical Chemistry and Electrochemistry Leibniz University Hannover Callinstraße 3A D‐30167 Hannover Germany; ^2^ Faculty of Advanced Science and Technology Kumamoto University 2‐39‐1 Kurokami Chuo‐ku Kumamoto 860‐8555 Japan; ^3^ Institute of Production Engineering and Machine Tools Leibniz University Hannover An der Universität 2 D‐30823 Garbsen Germany; ^4^ Fraunhofer Institute for Microstructure of Materials and Systems IMWS Walter‐Hülse‐Straße 1 D‐06120 Halle Germany; ^5^ Department of Materials and Earth Sciences Technical University of Darmstadt Peter‐Grünberg‐Straße 2 D‐64287 Darmstadt Germany

**Keywords:** anisotropy, magnetic orientation, neodymium nickel oxide, oxygen transport membranes, Ruddlesden–Popper oxides, textured ceramics

## Abstract

Textured Nd_2_NiO_4+_ *_δ_* bulk ceramic membranes are fabricated via slip casting in a 0.9 T magnetic field generated by neodymium magnets. This process aligns the oxide grains with their easy‐magnetization *c*‐axis parallel to the applied magnetic field. Depending on the magnetic field's direction relative to the slip casting, grains orient either with their *a*,*b*‐plane or *c*‐axis parallel to the normal direction of the disk‐shaped ceramic, thus aligning with the oxygen permeation direction. Without the magnetic field, a non‐textured bulk membrane is formed. The microstructure and texture of the ceramic membranes are meticulously analyzed using advanced techniques, including X‐ray diffraction, scanning and transmission electron microscopy, as well as related methods. Evaluation of the texturing effect on the oxygen permeation performance shows that the *a*,*b*‐plane textured Nd_2_NiO_4+_ *_δ_* bulk membrane achieves the highest oxygen permeation fluxes between 1023–1223 K. Additionally, it demonstrates impressive CO₂ stability, maintaining effective performance for at least 140 h due to preferential oxygen transport along the *a*,*b*‐plane. These characteristics make Nd_2_NiO_4+_ *_δ_* an auspicious material for industrial applications as an oxygen transport membrane, outperforming more susceptible perovskite‐based materials. Magnetic alignment thus proves to be an effective method for achieving membrane texturing, enabling precise regulation of oxygen transport properties.

## Graphical abstract

Grain orientation in Nd_2_NiO_4+_ *_δ_* ceramic membranes, achieved using magnetic fields, enables precise tuning of oxygen transport properties. This study presents an Nd_2_NiO_4+_ *_δ_* bulk membrane with grains aligned with their *a*,*b*‐plane parallel to the oxygen permeation direction, produced by a neodymium magnet. The textured ceramic demonstrates high oxygen permeation efficiency and exceptional CO₂ stability, highlighting its potential for cutting‐edge industrial applications.

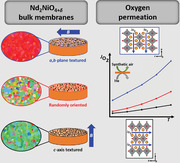

## 1 Introduction

Recently, mixed ionic‐electronic conductors (MIECs) have attracted growing interest due to their role as oxygen‐transporting membranes (OTMs) in various applications.^[^ ^1^ ^]^ These include the production of high‐purity oxygen,^[^ ^2^, ^3^ ^]^ integration into petrochemistry processes such as the oxidative coupling of methane to ethylene and/or ethane,^[^ ^4^, ^5^ ^]^ utilization in CO_2_ capture and storage technologies,^[^ ^6^, ^7^, ^8^ ^]^ and as cathode materials in solid oxide fuel cells (SOFCs)^[^ ^9^ ^]^ or rechargeable lithium–air batteries.^[^ ^10^ ^]^ Single‐phase alkaline earth metals‐containing perovskite‐type (ABO_3_) based OTMs are known for their superior oxygen permeation, attributed to their excellent mixed ionic and electronic conductivity.^[^ ^11^, ^12^, ^13^, ^14^, ^15^ ^]^ These materials typically achieve oxygen permeation exceeding 1 mL min⁻^1^ cm⁻^2^ at 1173 K for 1 mm thick membranes.^[^ ^11^, ^13^ ^]^ The oxygen permeation performance can be further enhanced by adopting a hollow‐fiber membrane configuration.^[^ ^11^, ^16^, ^17^ ^]^ However, the practical application of the perovskite‐type OTMs is limited due to their poor stability in CO_2_‐containing atmospheres. The exposure to CO_2_ leads to the formation of alkaline earth metal carbonate layers, significantly reducing the oxygen flux.^[^ ^3^, ^13^, ^18^, ^19^, ^20^, ^21^, ^22^ ^]^

In contrast, the perovskite‐related Ruddlesden–Popper (RP) layered oxides Ln_2_NiO_4+_ *_δ_* (Ln = La, Nd or Pr) with K_2_NiF_4_ structure represent promising candidates for OTM development due to their high long‐term chemical stability under CO_2_ atmosphere and substantial mixed ionic‐electronic conductivity.^[^ ^11^, ^13^, ^23^, ^24^, ^25^, ^26^ ^]^ Nevertheless, their oxygen permeation is inferior to that of the perovskite‐derived OTMs. One of the first members of the RP oxide series, which is the focus of our research is Nd_2_NiO_4+_ *_δ_* (NNO). Its crystal structure consists of an alternating stacking of cubic perovskite NdNiO_3_ layers and NdO rock‐salt layers along the crystallographic *c*‐axis.^[^ ^27^, ^28^, ^29^, ^30^, ^31^ ^]^ Moreover, structural strains caused by the large interlayer lattice mismatch are present. These tensions are relieved through the incorporation of interstitial oxygen ions (O^2−^) into the rock‐salt layers, leading to oxygen hyperstoichiometry *δ*, which typically ranges from 0.22 to 0.28.^[^ ^29^, ^30^, ^32^, ^33^, ^34^, ^35^, ^36^ ^]^ Depending on *δ* and the temperature, NNO assumes either an orthorhombic or a tetragonal structure. The tetragonal phase can display an *I*4/*mmm* or a *P*4_2_/*ncm* symmetry, while the orthorhombic structure can be linked to the *Fmmm* or *Bmab* space group.^[^ ^37^, ^38^, ^39^, ^40^ ^]^ Oxygen transport in NNO is highly anisotropic, primarily occurring in the rock‐salt layers along the *a*,*b*‐plane via a two‐dimensional 2D push‐pull interstitialcy diffusion mechanism. Here, an interstitial O^2−^ occupies an apical O^2−^ site in the NiO_6_ octahedron after the apical O^2−^ has migrated to an unoccupied interstitial site.^[^ ^27^, ^29^, ^41^, ^42^, ^43^, ^44^, ^45^, ^46^ ^]^ Oxygen migration along the *c*‐axis is less prominent and involves the movement of oxygen vacancies through the perovskite layers.^[^ ^41^, ^42^, ^43^, ^44^, ^45^, ^46^, ^47^ ^]^ The fabrication of oriented NNO single crystals by Bassat et al.^[^ ^48^ ^]^ has opened new avenues for harnessing the anisotropic nature of this material to precisely tune the oxygen transport properties along the *a*,*b*‐plane or *c*‐axis. This is demonstrated by the oxygen bulk diffusion coefficients D* being approximately three orders of magnitude larger and the oxygen surface exchange coefficients k* being about 1–1.5 orders of magnitude higher in the *a*,*b*‐plane compared to the *c*‐axis, in the temperature range of 723–973 K.^[^ ^48^ ^]^ Later, Yamada et al.^[^ ^49^ ^]^ fabricated a (110)‐oriented NNO epitaxial film (14 nm thickness) on the (100)‐surface of Y_2_O_3_‐stabilized ZrO_2_ electrolyte using pulsed laser deposition. The NNO epitaxial film showed enhanced performance as an oxygen cathode for SOFCs due to its preferential orientation along the *a*,*b*‐plane.

On the other hand, in a polycrystalline membrane containing randomly oriented grains, the anisotropic character of NNO is not fully exploited. Therefore, texturing the polycrystal offers the potential to regulate the oxygen diffusion in the *a*,*b*‐plane or *c*‐axis, potentially achieving properties comparable to those of the corresponding single crystals. Well‐developed texturing techniques for polycrystalline materials include the rolling assisted biaxially textured substrates process,^[^ ^50^, ^51^ ^]^ powder aerosol deposition,^[^ ^52^, ^53^ ^]^ powder‐in‐tube,^[^ ^50^, ^54^, ^55^ ^]^ templated grain growth (TGG),^[^ ^50^, ^56^, ^57^ ^]^ and reactive templated grain growth.^[^ ^50^, ^58^ ^]^ These methods can be combined with advanced sintering processes such as hot pressing,^[^ ^59^, ^60^ ^]^ spark plasma sintering (SPS),^[^ ^61^, ^62^ ^]^ spark plasma texturing,^[^ ^63^, ^64^ ^]^ or pressure‐assisted sintering^[^ ^65^ ^]^ to produce highly textured, dense ceramics. To date, research has primarily focused on the texturing of optical,^[^ ^50^, ^66^ ^]^ thermoelectric,^[^ ^50^, ^67^, ^68^ ^]^ and piezoelectric polycrystalline materials.^[^ ^50^, ^57^, ^69^, ^70^, ^71^ ^]^ This concept can potentially be extended to MIECs‐based OTMs. For example, in a previous publication, a TGG‐derived method was applied to the RP oxide La_2_NiO_4+_ *_δ_* to fabricate textured OTMs using plate‐like La_2_NiO_4+_ *_δ_* template particles from a molten flux synthesis.^[^ ^72^, ^73^ ^]^ During cold pressing and subsequent pressureless sintering, the template particles remained oriented, with their *c*‐axis preferentially parallel to the pressing direction. Moreover, the production of nanostructured OTMs using the SPS technique from La_2_NiO_4+_ *_δ_* nanorods obtained by reverse microemulsion has been reported.^[^ ^74^ ^]^ The high abundance of (1$\bar{1}$0)‐type facets on the sides of the orthorhombic nanorods, which favor surface oxygen exchange, led to superior oxygen permeation in the nanorod‐derived membranes. This improvement is particularly notable between 1023–1123 K, outperforming their nanoparticle‐based counterparts.^[^ ^74^ ^]^

Texturing in polycrystalline materials can also be achieved by magnetically orienting the particles in the starting powder through the application of an external magnetic field. This requires the material to have an anisotropic crystal structure with anisotropic magnetic susceptibilities.^[^ ^50^, ^75^, ^76^ ^]^ The driving force for the magnetic particle orientation is defined by Equation (1) as follows:

(1) $$\Delta E=\frac{\Delta \chi VB^{2}}{2\mu_{0}}$$

where Δ*E* is the magnetic anisotropic energy, Δ*χ*  = *χ*_*a,b*_ −  *χ*_*c*_ is the difference in magnetic susceptibility in the *a*,*b*‐plane (*χ*_*a,b*_) and the *c*‐axis (*χ*_*c*_), *V* is the volume of each particle, *B* is the applied magnetic field, and *μ*_0_ is the vacuum permeability. The particle alignment is given when Δ*E* is higher than *k*_B_ *T*, with *k*_B_ being the Boltzmann constant, and *T* the absolute temperature.^[^ ^76^, ^77^, ^78^ ^]^ The development of textures in ceramic systems such as Al_2_O_3,_^[^ ^78^, ^79^ ^]^ TiO_2,_^[^ ^76^ ^]^ TiB_2,_^[^ ^80^ ^]^ AlN,^[^ ^81^ ^]^ and BaTiO_3_^[^ ^82^ ^]^ using magnetic fields has been extensively investigated. NNO presents an anisotropic layered crystal structure, displaying different molar magnetic susceptibilities in the *a*,*b*‐plane ($\chi_{a,b}^{m}$  =  2.42 × 10⁻^3^ cm^3 ^mol⁻^1^) and along the *c*‐axis ($\chi_{c}^{m}$   = 0.86 × 10⁻^3^ cm^3 ^mol⁻^1^).^[^ ^83^ ^]^ Assuming Δ*χ* ^*m*^ = 1.56 × 10⁻^3^ cm^3 ^mol⁻^1^, and that the particles are polyhedral with their size in the micrometer range, NNO crystals can be oriented in a magnetic field of 0.8–2.0 T.^[^ ^77^, ^83^, ^84^ ^]^ Murata et al.^[^ ^85^ ^]^ studied the magnetic behavior of NNO and determined by X‐ray diffraction (XRD), that the easy‐magnetization axis of this oxide is the *c*‐axis. They used these findings to produce an NNO cathode layer oriented with the *a*,*b*‐plane perpendicular to the surface of a Gd_0.1_Ce_0.9_O_1.95_ solid electrolyte in a 0.9 T magnetic field. The cathode material showed an improved performance for low‐temperature operating SOFC (LT‐SOFC), attributed to the *a*,*b*‐plane alignment. Furthermore, magnetic orientation was applied to other RP oxides, such as La_2_NiO_4+_ *_δ_* and Pr_4_Ni_3_O_10_, achieving similar enhancements in performance as electrodes for LT‐SOFCs.^[^ ^86^, ^87^, ^88^ ^]^ Zhao et al.^[^ ^84^ ^]^ also utilized the magnetic method to fabricate textured dense NNO films with micrometer‐scale thicknesses ranging from 50 to 162 µm on a porous support, resulting in asymmetric membranes. The grains within the layers were oriented with their *a*,*b*‐plane perpendicular to the applied magnetic field of 0.81 T. The oxygen permeation flux of the disk‐shaped asymmetric ceramic with a 162 µm thick dense NNO film was roughly tripled in comparison to a polycrystalline bulk membrane when subjected to He sweeping at 1223 K. Besides, the membrane demonstrated excellent long‐term stability in CO_2_ for ≈120 h, maintaining an oxygen permeation flux of 1.4 mL min⁻^1^ cm⁻^2^ at 1223 K.^[^ ^84^ ^]^ This marked a crucial improvement over CO_2_‐susceptible perovskite‐based OTMs.

In this work, the concept of magnetic orientation was extended to the development of dense bulk OTMs by aligning NNO crystals in a magnetic field of 0.9 T by slip casting. During the orientation process, the particles were aligned such that their *a*‐ and *b*‐axes, as well as the *a*,*b*‐plane or *c*‐axis, were parallel to the normal direction of the disk membrane, which coincides with the direction of oxygen permeation. Following isostatic cold pressing and pressureless sintering, textured bulk membranes are obtained, exhibiting a preferred grain orientation with the *a*,*b*‐plane or *c*‐axis. Furthermore, a polycrystalline NNO membrane prepared outside the magnetic field was used as a reference. Various characterization methods were applied for the texture and microstructural analysis and the impact of the texturing on the oxygen permeation properties was thoroughly examined, discussed, and compared to those of the non‐texturized sample.

## 2 Results and Discussion

### 2.1 Magnetic Alignment of Nd_2_NiO_4+*δ*_ Particles and Membrane Preparation

**Figure 1:**
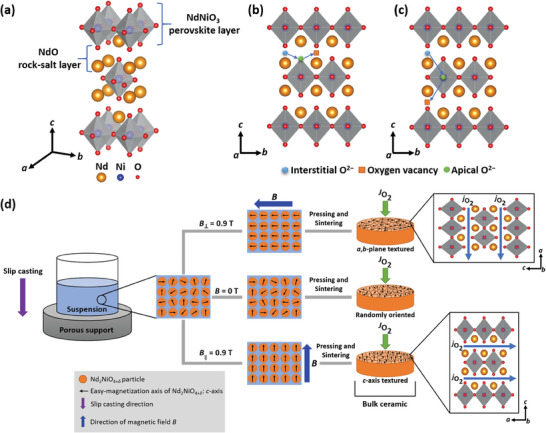
a) Orthorhombic crystal structure of Nd_2_NiO_4+_ *_δ_* (NNO) in the *Fmmm* space group. Oxygen transport mechanism along the b) *a*,*b*‐plane and c) *c*‐axis of NNO.^[^ ^27^, ^29^, ^41^, ^42^, ^43^, ^44^, ^45^, ^46^, ^47^ ^]^ d) Schematic representation illustrating the preparation of NNO ceramic bulk membranes by slip‐casting in or outside of a magnetic field *B*. The green arrows indicate the direction of oxygen permeation flux $J_{O_{2}}$ through the membrane, while the blue arrows depict the oxygen diffusion path $j_{O_{2}}$ through NNO.

For the preparation of the ceramic bulk membranes, NNO powder obtained through a solid‐state route was employed. The XRD pattern of the powder in Figure S1 (Supporting Information) displays the presence of orthorhombic NNO in the *Fmmm* space group as a single phase. This observation is consistent with earlier studies on NNO, where the orthorhombic *Fmmm* phase was detected at room temperature and in an oxygen‐rich atmosphere, as evidenced by powder XRD or powder neutron diffraction.^[^ ^35^, ^36^, ^39^, ^40^, ^89^, ^90^ ^]^ Scanning electron microscopy (SEM) analysis, presented in Figure S1b (Supporting Information), reveals polyhedral NNO particles with an average size of 0.63 µm (Figure S1c, Supporting Information), which tend to form agglomerates. In **Figure**  **1**, both the crystal structure of NNO in its orthorhombic form (space group: *Fmmm*) and a schematic representation of the ceramic membrane preparation are given. Apart from the crystal structure (Figure  1a), the possible oxygen diffusion mechanisms in the *a*,*b*‐plane and *c*‐axis are indicated by arrows in Figure  1b,c, respectively. Oxygen transport in the rock‐salt layers along the *a*,*b*‐plane is defined by the interstitialcy mechanism, while in the perovskite layers in the *c*‐axis, it occurs via the vacancy diffusion mechanism.^[^ ^29^, ^41^, ^42^, ^44^, ^91^ ^]^ The preparation of the ceramic membranes (Figure  1d) began with the magnetic alignment of the NNO particles by slip casting. The suspension containing the NNO powder was cast into a bottomless cylindrical vessel placed on a porous support. During slip casting, the suspension solidified through the gradual removal of the solvent, driven by the capillary pressure exerted by the porous support.^[^ ^75^ ^]^ Concurrently, the NNO particles were deposited onto the surface of the support. A magnetic field *B* of 0.9 T was applied to the suspension during slip casting, ensuring that the easy‐magnetization axis of NNO, that is, the *c*‐axis, aligned parallel to the direction of *B*.^[^ ^84^, ^85^ ^]^ Moreover, depending on the orientation of the applied *B* relative to the gravitational sedimentation of the slip casting direction, three particle orientation scenarios can be distinguished: i) due to the random rotation around the easy‐magnetization *c*‐axis, the particles oriented with their *a*‐ and *b*‐axes, as well as the *a*,*b*‐plane perpendicular to the applied *B* (= *B*_⊥_). The disk‐shaped green compact was then densified through cold isostatic pressing, whereby the particle orientation was retained, and subsequently sintered in air pressurelessly. This resulted in a textured bulk membrane where the *a*‐ and *b*‐axes, and *a*,*b*‐plane were primarily parallel to the normal ceramic direction and aligned with the oxygen permeation flux $J_{O_{2}}$ direction. Consequently, the preferential oxygen diffusion path $j_{O_{2}}$ through NNO along the *a*,*b*‐plane runs parallel to the $J_{O_{2}}$ direction. ii) When no *B* was applied, the NNO particles were randomly oriented, producing a polycrystalline bulk ceramic. iii) When *B* was applied parallel (= *B*_∥_) to the casting direction, the NNO crystals and, thus, the membrane grains were aligned with their *c*‐axis parallel to the normal ceramic and $J_{O_{2}}$ direction. This means, that $j_{O_{2}}$ occurs perpendicular to the $J_{O_{2}}$ direction. In the following, the textured NNO bulk ceramics will be referred to as either *a*,*b*‐plane textured or *c*‐axis textured membranes.

### 2.2 Microstructural and Textural Analysis of Nd_2_NiO_4+_ *_δ_* Membranes

**Figure 2:**
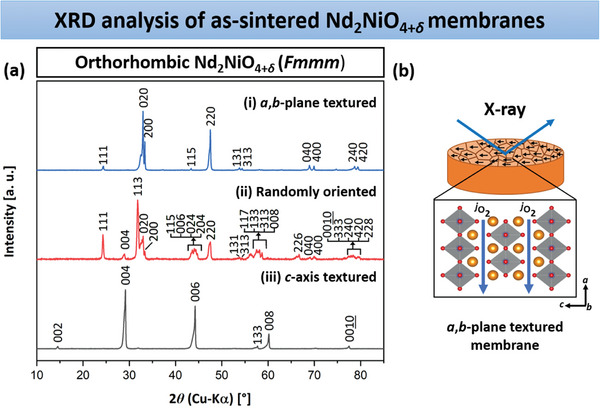
a) Room temperature XRD patterns of the NNO bulk membranes, obtained with or without magnetic alignment (*B* = 0.9 T): i) *a*,*b*‐plane textured; ii) randomly oriented, and iii) *c*‐axis textured. The XRD patterns were recorded from the surface of the ceramics, as shown in (b).

Microstructural characterization and textural analysis of the disk‐formed NNO ceramic membranes produced by magnetic slip casting were conducted using XRD, SEM, and transmission electron microscopy (TEM). For phase identification, XRD patterns (**Figure**  **2**a) were recorded from the surface of the three NNO bulk ceramics (Figure  2b). The assignment of the reflections indicates that NNO is present in all membranes as a single‐phase orthorhombic structure with the *Fmmm* space group, consistent with the XRD pattern of the starting powder (Figure S1a, Supporting Information). Energy dispersive X‐ray spectroscopy (EDXS) analyses of the bulk membranes, shown later, reveal the presence of NiO as a minor phase. This phase could not be detected by XRD, indicating that its amount is negligible. Compared to the randomly oriented sample, the other membranes exhibit a strong texturing either along the *a*,*b*‐plane or *c*‐axis. For the *a*,*b*‐plane textured NNO ceramic, the intensities of *h*00, 0*k*0, or *hk*0 reflections are notably pronounced, while for the *c*‐axis textured oxidic specimen, the 00*l* reflections exhibit high intensity. The 113 reflection, which represents the primary reflection in the polycrystalline ceramic, is absent in the textured membranes, suggesting a substantial degree of texturing induced by magnetization. Nevertheless, it is noteworthy that the *a*,*b*‐plane textured bulk ceramic displays additional low‐intensity reflections not corresponding to the *a*‐axis, *b*‐axis, or *a*,*b*‐plane, such as 111, 115, and 131. In contrast, the *c*‐axis‐oriented sample presents only one secondary reflection, 133, which does not match the *c*‐axis. Next, Lotgering orientation factor *f* was calculated from the XRD patterns in the 2*θ* range of 10 to 85° using Equation (2).^[^ ^92^, ^93^ ^]^ The Lotgering factor *f* quantitatively assesses the texturing degree in each ceramic sample and ranges from 0 to 1, where 0 denotes a random grain orientation and 1 signifies a perfectly aligned grain structure.^[^ ^94^ ^]^ The starting NNO powder (*f* = 0) served as the reference for calculating the degree of texturing in the membranes. While the polycrystalline ceramic sample has an *f* = 0.05, the *f* values for the *a*,*b*‐plane and *c*‐axis textured bulk membranes amount to 0.92 and 0.99, respectively. **Table** **1** summarizes the *f* values along with other key characteristics of the ceramic samples. The XRD results obtained in this work corroborate earlier findings, confirming that the easy‐magnetization axis of NNO is the *c*‐axis.^[^ ^84^, ^85^ ^]^ This conclusion is further supported by the calculation of magnetic anisotropic energy (Δ*E*) for all three samples, using Equation (1) and alongside data from Table 1 and Table S1 (Supporting Information). A detailed description of the calculation of ΔE is provided on pages 2 and 3 of the Supporting Information. A lower Δ*E* of 2.99 10⁻^16 ^J is required for the magnetization of the *c*‐axis, which explains the extremely strong 00*l* texturing and the *f* value close to 1. In contrast, the *a*‐ and *b*‐axes are the hard‐magnetization axes of this RP oxide, demanding a higher Δ*E* of 13.80 10⁻^16 ^J for effective magnetization in these directions.^[^ ^50^, ^95^ ^]^ Consequently, due to the more difficult magnetization, the *a*,*b*‐plane textured sample exhibits not only the highly intense *h*00, 0*k*0, and *hk*0 reflections but also weaker reflections, such as 111 and 115, which are not associated with the *a*‐ or *b*‐axes or the *a*,*b*‐plane. A Δ*E* value of 4.17 10⁻^16 ^J was estimated for the non‐textured NNO ceramic, which falls between the values of the *a*,*b*‐plane‐ and *c*‐axis‐textured samples. This outcome is expected, as the non‐textured sample contains randomly oriented grains along the *a*‐, *b*‐, and *c*‐axes, leading to an average magnetic anisotropic energy.

**Table 1:** Overview of the microstructural characteristics of NNO ceramic membranes.

NNO ceramic membrane	Oxygen hyper‐stoichiometry *δ*	Relative density [%]	Experimental density [g cm^−3^]	Grain size [µm]	Lotgering factor *f*	XRD pole figures MRD		
						111	004	220
*a*,*b*‐plane textured	0.25	98.42	7.43	5.72	0.92	4.17	3.12	7.43
randomly oriented	0.24	98.83	7.46	4.44	0.05	1.87	1.77	2.88
*c*‐axis textured	0.26	98.26	7.42	4.86	0.99	3.24	25.33	2.79

The influence of magnetic field strength on the level of texturing in NNO ceramics has been explored in previous studies.^[^ ^84^, ^85^ ^]^ For instance, Murata et al.^[^ ^85^ ^]^ prepared *a*,*b*‐plane and *c*‐axis textured NNO bulk ceramics in the orthorhombic structure by employing slip casting in a 5 T magnetic field created by a superconducting magnet. The XRD patterns of their *a*,*b*‐textured samples exhibited strong 020, 200, and 220 reflections, indicating grain orientation along the *a*‐ and *b*‐axes, as well as within the *a*,*b*‐plane of NNO. Additionally, Murata et al.^[^ ^85^ ^]^ reported *f* values of 0.87 for the *a*,*b*‐plane and 0.95 for the *c*‐axis textured NNO ceramics, which are slightly lower than the *f* values of 0.92 and 0.99, respectively, obtained in this study. Beyond the magnetic field strength, several other parameters significantly affect the degree of magnetic particle orientation, including the composition of the slip casting suspension, the dispersion degree of the NNO particles within the suspension, the NNO crystal size, and the pressing and sintering conditions for producing the textured ceramic samples. These factors have been optimized in this work and therefore differ from those reported by Murata et al.,^[^ ^85^ ^]^ which may also explain the variation in the degree of texturing. Consequently, increasing the magnetic field does not substantially impact the particle alignment and thus the degree of texturing in the NNO membranes. Calculated anisotropic magnetic energies (see Table S1, Supporting Information) also imply that particle orientation along the *a*,*b*‐plane requires higher energy than alignment along the *c*‐axis in NNO. Conversely, Zhao et al.^[^ ^84^ ^]^ employed a 0.81 T magnetic field to develop *a*,*b*‐plane textured dense NNO films in asymmetric membranes with enhanced oxygen permeability via drop casting. The relatively low magnetic field strength resulted in a moderate grain orientation with *f* = 0.31, pointing to the need for a stronger field to achieve enhanced grain alignment. Despite the moderate texturing degree, a significant improvement in oxygen permeation was observed compared to the non‐textured variant. Zhao's findings, along with a comparison to our results, are discussed in detail later. Overall, our research work demonstrates that a 0.9 T magnetic field is sufficient to produce highly textured NNO ceramic membranes. This is substantiated by the relationship between the minimum particle size necessary for magnetic orientation and the magnetic field strength, as proposed by Kimura et al.^[^ ^77^ ^]^ For the NNO powder investigated, with a minimum particle size of 300 nm (see Figure S1c, Supporting Information) and a Δ*χ* = 10^−5^, a magnetic field strength of 0.2 T is the maximum required for magnetic alignment, which is lower than the 0.9 T field applied in this work.

The oxygen hyperstoichiometry *δ* of the NNO membranes measured by hot gas extrusion analysis ranged from 0.24 to 0.26 (see Table 1). The calculated *δ* values agree with the literature values (0.13 ≤ *δ *≤ 0.25)^[^ ^35^, ^36^, ^40^, ^89^ ^]^ and are characteristic of the existence of an orthorhombic crystal structure with *Fmmm* symmetry at room temperature, as confirmed by the XRD measurements in Figure  2.

**Figure 3:**
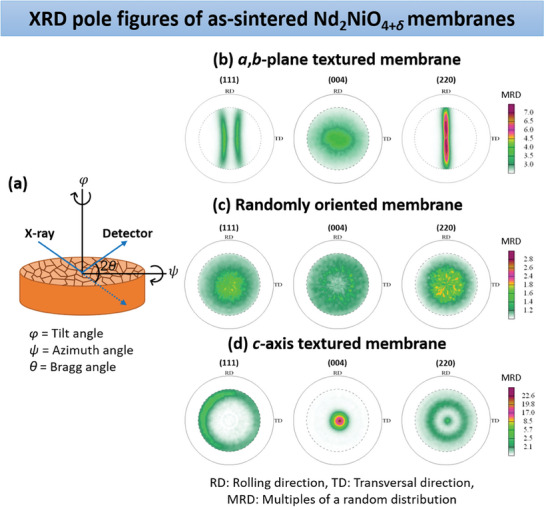
a) Schematic representation of the pole figure measurement principle on a ceramic sample. XRD pole figures of the (111), (004), and (220) planes with MRD intensity scale in a.u. obtained from b) NNO ceramic textured within the *a*,*b*‐plane, c) randomly oriented membrane, and d) *c*‐axis textured bulk ceramic. Note different intensity scales for MRD.

Further quantitative analysis of the texturing level was conducted by recording XRD pole figures from the (111), (004), and (220) planes of both textured ceramics and the polycrystalline sample. In the pole figure configuration (**Figure**  **3**a), the sample was tilted (tilt angle *φ* = 0–70°). At each *φ* position, the ceramic was rotated about its normal direction (azimuth angle *ψ* = 0–360°), while XRD signals were simultaneously acquired by the detector at a fixed 2*θ* position.^[^ ^96^, ^97^ ^]^ To estimate the texturing degree in the ceramic specimens, the pole figures were normalized to units of multiples of a random distribution (MRD) using LaboTex software. The MRD value can adopt values between 1 (=no texturing) and infinite (=single crystal). For a textured ceramic, a higher MRD value reflects a greater degree of texturing.^[^ ^65^, ^73^, ^98^, ^99^, ^100^ ^]^ The maxima of the MRD values for all samples are listed in Table 1. A pole figure is defined as a 2D representation of grain orientation for a specific crystallographic lattice plane.^[^ ^101^ ^]^ In a (100) pole figure, the poles of the (100) planes in the grains are oriented to match the axes defined by the rolling, transversal, and normal directions, which are specific to the sample's coordinate system.^[^ ^102^, ^103^ ^]^ For the *a*,*b*‐plane oriented NNO membrane, the (220) pole figure depicted in Figure  3b exhibits a higher intensity with an MRD value of 7.43, compared to MRD values of 4.17 and 3.12 for the (111) and (004) pole figures, respectively. This indicates a pronounced degree of (*hk*0) texturing in the sample. The stripe pattern in the (220) pole figure indicates that the (220) lattice planes in the grains are preferentially aligned in the *a*,*b*‐plane but show a random and spread orientation around the *c*‐axis. Although the 020 reflection is the most dominant in the XRD diffractogram of the NNO ceramic (Figure  2a) with grains *a*,*b*‐plane oriented, and would therefore be expected to yield the highest MRD value, no pole figure was taken for this plane. This is because the measurement method cannot differentiate between the 020 and 200 reflections due to their similar 2*θ* positions and the limitations of the instrument.^[^ ^84^ ^]^ Interestingly, the randomly oriented NNO ceramic (Figure  3c) also reveals a modest degree of (*hk*0) texturing, with the (220) pole figure having a maximum MRD value of 2.88. Conversely, the (111) and (004) pole figures present similarly low MRD values of 1.87 and 1.77, respectively. The pronounced (00*l*) texturing in the *c*‐axis oriented sample is highlighted by the exceptionally high intensity of the (004) pole figure in Figure  3d. Its maximum MRD value is 25.33, approximately eight to nine times greater than the MRD values for the (111) and (220) pole figures, which are 3.24 and 2.79, respectively. The (220) pole figure of this ceramic specimen displays a central spot and rings with low intensity. This suggests that while the grains are *c*‐axis oriented, the (220) planes are randomly distributed around this axis, resulting in relatively weak overall texture strength. In accordance with the calculated Lotgering factor *f* (refer to Table 1) from the XRD diffractograms in Figure  2a, the pole figure measurements further confirm the outstanding grade of texturing in the NNO ceramic membranes, with grains oriented with their *a*,*b*‐plane or *c*‐axis, achieved by the magnetic alignment method.

**Figure 4:**
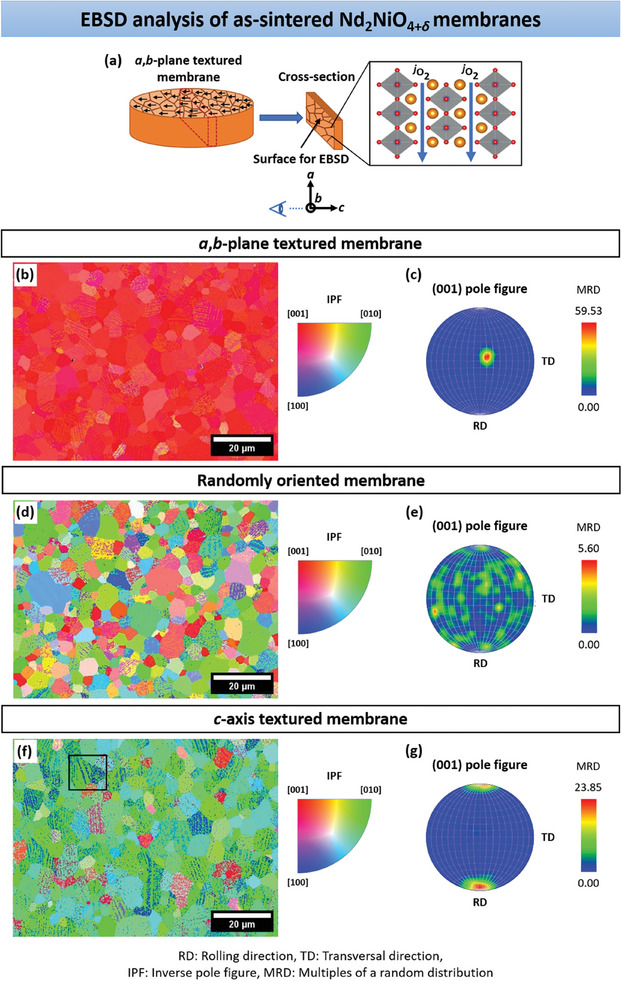
a) Schematic diagram illustrating the region of interest for EBSD analysis in the cross‐section of the *a*,*b*‐plane textured NNO ceramic. In the cross‐section, the grain orientation is along the *c*‐axis of NNO. Cross‐sectional EBSD orientation maps and (001) pole figures of b,c) *a*,*b*‐plane textured, c,d) randomly oriented, and f,g) *c*‐axis textured NNO membranes. The EBSD maps are colored according to the inverse pole figure (IPF) color key. Each color in the IPF corresponds to a specific crystallographic direction. The degree of texture in the pole figures is represented by MRD values on the intensity scale bar (in a.u), with a color scheme distinct from that of the IPF color key. Different intensity scales are used for the MRD values of each sample. The inset in Figure  4. f) highlights two grains with [010] and [100] orientations.

Electron backscatter diffraction (EBSD), an SEM‐based technique, was applied to further characterize the texturing in the NNO ceramic samples. Vibratory‐polished cross‐sections were prepared and employed to ensure high‐resolution imaging and improved contrast in all SEM experiments. As shown in **Figure**  **4**a, for the *a*,*b*‐plane textured bulk sample, the EBSD analysis is conducted parallel to the *c*‐axis of the oriented grains. Conversely, in the *c*‐axis oriented ceramic, the *a*‐ and *b*‐axes, or the *a*,*b*‐plane of the grains, are aligned parallel to the EBSD analysis surface. To validate this, XRD patterns of the membrane cross‐sections were recorded (see Figure S2, Supporting Information). Figure  4b–g presents the EBSD orientation maps together with the (001) pole figures for the ceramic membrane cross‐sections. In the EBSD maps, each color represents a grain orientation along a specific crystallographic direction, following the inverse pole figure (IPF) color scheme. The EBSD orientation maps are based on the EBSD band contrast images, which indicate the signal intensity of the Kikuchi bands and are shown as grayscale components.^[^ ^104^, ^105^, ^106^, ^107^ ^]^ These band contrast maps are displayed in Figure S3a,c,e (Supporting Information), which also includes the (100) and (010) pole figures for each sample. The cross‐sectional EBSD map of the *a*,*b*‐plane textured NNO membrane in Figure  4b displays preferential texturing along the [001] direction, indicated by the intense red color. This is reflected in the prominent peak near the center of the (001) pole figure, which has a maximum MRD value of 59.53 (Figure  4c), while the (100) and (010) pole figures (Figure S3b, Supporting Information) show no significant texturing. Note that the color coding of the MRD intensity scale bar differs from that of the IPF color key. The cross‐section of the non‐textured ceramic exhibits a colorful EBSD map (Figure  4d), demonstrating a random orientation distribution of the grains across the three crystallographic directions. The pole figures for the (010), (100), and (001) lattice planes (Figure  4e; Figure S3d, Supporting Information) confirm the absence of distinctive grain orientation in the sample, supported by the relatively low MRD value of 5.53. Remarkably, for the *c*‐axis textured NNO ceramic, it was found that the grains in the cross‐section exhibit orientations between [100] and [010], as evidenced by the extensive blue‐green coloration, as well as their mixtures, in the EBSD map presented in Figure  4f. This results in the yellow‐red marked areas at the top and bottom of the (001) pole figure (Figure  4g) and the green region near the center of the (100) and (010) pole figures in Figure S3f (Supporting Information), with a maximum MRD value of 23.85. This observation can be attributed to the magnetic properties of NNO. Given that the *c*‐axis is the easy‐magnetization direction in NNO, the magnetic field is less effective at precisely controlling the orientation of particles along either the *a*‐ or *b*‐axes.^[^ ^84^, ^85^ ^]^ Consequently, in the cross‐section of the *c*‐axis textured ceramic sample, a more pronounced grain orientation is observed along the *a*‐ and *b*‐axes, as well as within the *a*,*b*‐plane.

Adding to the calculated Lotgering factor *f* (Table 1) from the XRD patterns depicted in Figure  2 and the XRD pole figures in Figure  3, the EBSD results further reinforce the strong grain orientation in the cross‐sections of the textured NNO ceramics relative to the polycrystalline sample. It is striking in the EBSD orientation maps (Figure  4b,d,f) that some grains exhibit multiple orientations. For instance, in the area marked by a rectangle in Figure  4f, grains are colored green and blue, indicating [010] and [100] orientations, respectively. The corresponding EBSD band contrast image highlights these different orientations by light‐dark stripes (see labeling in Figure S3e, Supporting Information).

Additional SEM studies were undertaken on the ceramic samples to enhance the understanding of the EBSD findings and to provide an in‐depth microstructure assessment. Both secondary electrons (SE) and backscattered electrons (BSE) SEM micrographs were captured from the vibratory‐polished and fractured membrane cross‐sections, as presented in Figures S4–S6 (Supporting Information). The membrane microstructure across all samples is characterized by the presence of polyhedral grains. Assuming a nearly round geometry, ≈100 grains from the SEM‐BSE micrographs in Figures S4b,S5b,S6b (Supporting Information) were evaluated using ImageJ,^[^ ^108^ ^]^ and the results are displayed as histograms (see Figures S4d,S5d,S6d, Supporting Information). The average grain size was estimated by fitting the histograms with a log‐normal distribution density function. Both the randomly and the *c*‐axis oriented NNO membranes have a similar average grain size of 4.44 and 4.86 µm, respectively. In comparison, the grains in the *a*,*b*‐plane textured NNO bulk ceramic are slightly larger, averaging 5.72 µm. A summary of the average grain size for all NNO samples is provided in Table 1. Besides, low porosity is evident in all SEM images, as reflected by the particularly high relative density values, which exceed 98% of the theoretical density for orthorhombic NNO of 7.55 g cm^−3^ (refer to Table 1).

**Figure 5:**
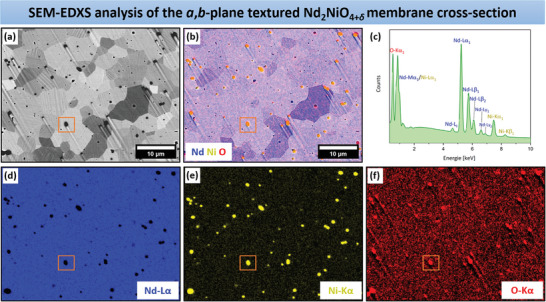
a) Backscattered electron channeling contrast SEM micrograph highlighting the selected area of the *a*,*b*‐plane textured ceramic for EDXS analysis. b) EDXS color mixing elemental map derived from (d–f). c) EDXS spectrum displaying the elemental composition from (a), and d,e) individual EDXS element maps of Nd (blue), Ni (yellow), and O (red). The NiO precipitations are emphasized by insets colored orange.

EDXS was utilized to investigate the sample composition and element distribution in the three disk‐shaped ceramics, with the results depicted in **Figure**  **5** and Figures S7,S8 (Supporting Information). For this end, SEM micrographs were collected using BSE channeling contrast from selected sample areas (Figure  5a; Figures S7a,S8a, Supporting Information). Similar to the EBSD studies (see Figure  4b,d,f; Figure S3a,c,e, Supporting Information), numerous grains exhibiting light‐dark stripes were identified. Additional SEM‐BSE images are available in Figure S9 (Supporting Information), further illustrating the presence of these striped grains. This phenomenon can be ascribed to the magnetic properties of NNO. Maity et al.^[^ ^38^ ^]^ reported antiferromagnetic behavior for this oxide below 150 K, which was corroborated by direct current magnetic susceptibility measurements. The observed stripes likely indicate magnetic domains (Weiss domains) present in antiferromagnetic materials such as NNO, where magnetic moments or electron spins are oppositely oriented within each domain when a magnetic field is applied.^[^ ^109^, ^110^, ^111^ ^]^ In the SEM‐BSE micrographs, the domain structure is visualized through the light‐dark contrast. Figure S9b (Supporting Information) presents a section of Figure S9a (Supporting Information), highlighting magnetic domains with different possible spin arrangements. Each domain exhibits distinct orientations (see, e.g., the marked area in Figure  4f), a pattern validated by EBSD analysis. Numerous reports in the literature document and confirm the observation of domain structures in magnetic materials using techniques such as SEM, TEM, and magnetic force spectroscopy.^[^ ^112^, ^113^, ^114^, ^115^, ^116^, ^117^ ^]^ Due to the higher sensitivity in a 40–60° degree angle of the magnetization axis to the primary beam, a forward scatter detector (FSD), as in EBSD, is usually used to visualize the type‐2 magnetic contrast (i.e., deflection of the BSE trajectories in the specimen by internal magnetic fields) in the SEM and make the aforementioned domains visible.^[^ ^112^, ^118^, ^119^, ^120^, ^121^ ^]^ Interestingly, in this work, magnetic domains are observed not only in EBSD geometry but also in conventional BSE geometry (i.e., semiconductor detector at the bottom of the objective pole piece.). This is likely attributed to the strong magnetic properties of NNO, which significantly influence the electron interactions and contrast, demonstrating that domain structures can be detectable even without the need for specialized FSDs.

The EDXS examination results on the ceramic samples are provided in Figure  5b–f and Figures S7b–e,S8b‐e (Supporting Information). For the *a*,*b*‐plane oriented NNO membrane, the EDXS spectrum shows the presence of Nd, Ni, and O. Both the colorful and individual EDXS element maps verify a homogeneous distribution of these elements in the investigated specimen section. Nevertheless, nickel‐rich regions are noticeable in the form of NiO, with an average size of ≈1.1 µm. In the SEM‐BSE micrograph (see insert in Figure  5a) the NiO presence is indicated by dark round areas. A similar occurrence was noted in the randomly oriented membrane (see Figure S7, Supporting Information). The *c*‐axis textured ceramic is also uniformly composed of Nd, Ni, and O, as affirmed by the EDXS results given in Figure S8 (Supporting Information). However, some areas with excess Nd were observed, existing as elemental Nd (≈1.0 µm) but in negligible amounts (see marking in the SEM‐BSE image in Figure S8a, Supporting Information). Statistically distributed NiO precipitations are also visible in this sample, as depicted in the cross‐sectional SEM micrographs in Figure S6a,b (Supporting Information), with some areas marked by rectangles. The EDXS data quantification demonstrates a stoichiometric Nd/Ni ratio of ≈2:1.1 across all membranes, as specified in Table S2 (Supporting Information). This suggests a marginal excess of nickel relative to neodymium in the samples. The formation of NiO precipitations in the RP oxides La_2_NiO_4+_ *_δ_*, NNO, and Pr_2_NiO_4+_ *_δ_* has been reported several times.^[^ ^74^, ^122^, ^123^, ^124^, ^125^, ^126^ ^]^ Bamburov et al.^[^ ^122^ ^]^ detected NiO using XRD and SEM‐EDXS in a Nd_1.95_NiO_4+_ *_δ_* ceramic membrane produced by conventional sintering, while Pikalova et al.^[^ ^123^ ^]^ identified the NiO phase in the Nd_1.5_Ca_0.5_NiO_4+_ *_δ_* system via TEM‐EDXS. Besides, Wahyudi et al.^[^ ^124^ ^]^ reported the presence of NiO as a secondary phase in the formation of Nd_2−_ *_x_* Sr*_x_* NiO_4+_ *_δ_* (*x* = 0.0, 0.1, and 0.5) single crystals. All these studies share the observation that NiO appears only as an impurity when stoichiometric Nd₂NiO_4+_ *_δ_* is not present. In the present work, the presence of NiO in the NNO ceramics can be attributed to the depletion of Nd in the main NNO phase due to elemental Nd formation, resulting in excess Ni that precipitates as NiO. The formation of NiO as a minor phase can be further understood in the content of the La─Ni─O phase diagram under air and at atmospheric oxygen pressure.^[^ ^127^ ^]^ At 1523 K, the sintering temperature for the NNO membranes, and with an Nd/Ni ratio of 2:1.1, the coexistence of NNO and NiO is anticipated. Ravkina et al.^[^ ^126^ ^]^ also noted NiO inclusions in La_2_NiO_4+_ *_δ_* ceramic membranes, but this impurity did not affect its oxygen transport properties due to its marginal content. Bamburov et al.^[^ ^122^ ^]^ prepared La_2−_ *_x_* NiO_4+_ *_δ_* (*x* = 0, 0.02, 0.05, and 0.10) ceramics and demonstrated via SEM‐EDXS that NiO segregations appeared at *x* > 0, with higher *x* values resulting in an increased NiO weight percentage ranging from 0.19 to 0.97 wt.%. Despite the NiO contamination, the oxygen permeation properties of the lanthanum‐deficient La_2_NiO_4+_ *_δ_* materials were comparable to those of cation‐stoichiometric La_2_NiO_4+_ *_δ_*. The NiO precipitates observed in the NNO membranes of this study were undetectable by XRD analysis (see Figure  2a; Figure S2b, Supporting Information), indicating a negligible amount; however, it was later identified and quantified by SEM‐EDXS. Using the SEM‐EDXS data from Table S2 (Supporting Information) and Equations S2 and S3 (Supporting Information), the weight percentage of NiO (wt.%_NiO_) in the ceramic samples was calculated and is presented in Table S3 (Supporting Information). The NNO membranes exhibit wt.%_NiO_ values ranging from 0.89 to 1.90%. Given the minor quantity of NiO, it is reasonable to conclude, in line with Bamburov et al.^[^ ^122^ ^]^ and Ravkina et al.^[^ ^126^ ^]^ that this impurity phase has no significant impact on the oxygen permeation fluxes.

**Figure 6:**
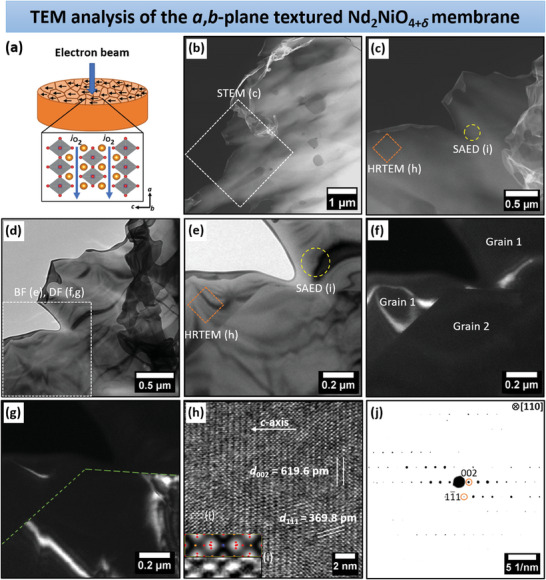
a) Chart highlighting the sample area of interest for the plan‐view TEM studies. b,c) STEM annular dark‐field micrographs at different magnifications displaying the investigated ceramic section. d,e) TEM bright‐field micrographs at different magnifications and f,g) corresponding dark field images based on a,b) illustrating the analyzed NNO grain. h) HRTEM micrograph, taken from the center of the marked area in c) or e), shows the lattice planes of orthorhombic NNO as well as the stacking order along the crystallographic *c*‐axis. i) Inset displays a magnified view of the region highlighted in h), along with a multislice simulated image, indicating the positions of neodymium, nickel, and oxygen, labeled with dots in orange, blue, and red, respectively. j) SAED pattern of the circled region in (c) or (e), with an oriented NNO grain along the [110] zone axis. To align with a), where the *c*‐axis is horizontal, both the HRTEM micrograph and the SAED pattern were rotated clockwise by ≈45°.

Further characterization of the microstructure and texture of the *a*,*b*‐plane textured NNO bulk ceramic membrane was carried out using TEM. The analysis focused on the plan‐view specimen, where the grains are aligned with their *c*‐axis perpendicular to the electron beam (**Figure**  **6**a). The scanning TEM (STEM) high‐angle annular dark field micrographs in Figure  6b,c, acquired at different magnifications, provide deep insights into the microstructure and identify the regions studied using high‐resolution TEM (HRTEM) and selected area electron diffraction (SAED). The TEM bright‐field (BF) micrographs in Figure  6d also highlight the selected sample area, with the magnified TEM‐BF image (Figure  6e) exhibiting the investigated NNO grain in direct contact with another grain. The corresponding TEM dark‐field (DF) micrographs in Figure  6f,g clearly illustrate the grain boundary between the two grains, as indicated by dashed lines in Figure  6g. Both the TEM‐BF and TEM‐DF images demonstrate the high crystallinity of the analyzed grain. Furthermore, the NNO grain was examined using HRTEM. The corresponding micrograph, presented in Figure  6h, was recorded from the center of the marked sample site shown in Figure  6c,e. The HRTEM analysis, illustrated in inset Figure  6i, reveals the stacking sequence along the *c*‐axis of NNO. This is complemented by a multislice simulated image, where neodymium, nickel, and oxygen atoms are indicated by orange, blue, and red spots, respectively. Moreover, the lattice spacings of the NNO phase were calculated as 369.8 pm between the (11) lattice planes and 619.6 pm between the (002) lattice planes, confirming the orthorhombic NNO structure. SAED was also performed on the same NNO grain (see Figure  6c,e) used for HRTEM (Figure  6h), oriented along the [110] zone axis. This produced a clear diffraction pattern with discrete diffraction, indicating a crystalline material. The diffraction spots allowed the assignment of Laue indices, as presented in Figure  6j. In the SAED experiment, the electron beam was perpendicular to the *c*‐axis, denoted by the (002) lattice plane, of the NNO grain along the [110] zone axis, which was also supported by the HRTEM results. This corresponds to a preferred orientation of the ceramic grains along the *a*,*b*‐plane (see Figure  6a), which is the most efficient oxygen transport pathway in NNO.

### 2.3 Oxygen Transport Properties of Nd_2_NiO_4+_ *_δ_* Membranes

**Figure 7:**
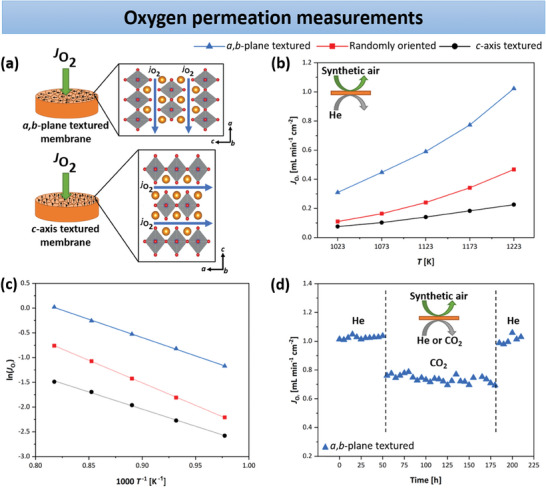
a) Concept of oxygen permeation measurement on the textured NNO disk membranes. In the oriented bulk ceramics, the grains are oriented with their *a*,*b*‐plane or *c*‐axis parallel to the oxygen permeation flux $J_{O_{2}}\;$ direction. b) Comparison of the oxygen permeation fluxes through the textured and non‐textured ceramics in a synthetic air/He gradient between 1023–1223 K. c) Arrhenius plots of the oxygen permeation used to determine the activation energy. d) Long‐term stability measurement of the *a*,*b*‐plane textured NNO bulk membrane in synthetic air/He and synthetic air/CO_2_ at 1223 K.

To explore the impact of texturing on the oxygen transport properties of dense NNO bulk membranes, oxygen permeation measurements were performed across a temperature range of 1023–1223 K. All ceramic membranes feature a thickness of 1 mm and met the gas‐tightness criterion, as their relative density exceeded 90%, the minimum requirement (see Table 1).^[^ ^34^, ^128^, ^129^ ^]^ The gas tightness of both the membranes and the sealant was further verified by gas chromatography, which detected the N_2_ concentration in the effluent stream. The relative oxygen leakage was determined to be less than 10% of the total oxygen concentration, validating the gas tightness of the ceramic membranes.^[^ ^130^ ^]^ As illustrated in **Figure**  **7**a, the grains in the magnetic field‐textured NNO membranes are oriented with their *a*,*b*‐plane or *c*‐axis parallel to the oxygen permeation flux $J_{O_{2}}$ direction. The results of the oxygen permeation measurements, presented in Figure  7b for all three bulk ceramic samples, demonstrate an increase in oxygen permeation flux with rising temperature. This behavior is characteristic of OTMs based on MIECs such as NNO.^[^ ^73^, ^84^, ^126^, ^129^, ^131^, ^132^ ^]^ Oxygen transport above 973 K is facilitated by defects in the crystal lattice, such as oxygen ion vacancies and/or interstitial oxygen ions. These defects favor the oxygen ion diffusion through the membrane and thereby contribute to the overall oxygen ion conductivity.^[^ ^11^, ^133^, ^134^ ^]^ Figure  7b clearly indicates that the *a*,*b*‐plane textured ceramic exhibits the highest oxygen permeation over the entire temperature range, followed by the polycrystalline sample. In contrast, the *c*‐axis textured NNO membrane has the lowest oxygen permeation. As outlined in the introduction, oxygen transport in NNO primarily occurs in the rock‐salt layers along the *a*,*b*‐plane, while oxygen diffusion in the *c*‐axis is less efficient due to the fully occupied oxygen sites in the perovskite layers. The oxygen transport within the *a*,*b*‐plane is explained by the interstitialcy mechanism (see Figure  1b), whereas along the *c*‐axis it takes place via the vacancy diffusion mechanism (Figure  1c).^[^ ^27^, ^29^, ^41^, ^42^, ^43^, ^44^, ^45^, ^46^, ^47^ ^]^ The oxygen permeation measurements in Figure  7b clearly reflect the anisotropic properties of NNO. Due to the random orientation of its grains, the non‐textured ceramic possesses average oxygen transport properties.

To allow a more accurate comparison of the oxygen permeation performance across the different bulk NNO membranes, **Table** **2** summarizes the oxygen permeation fluxes at the lowest (1023 K) and highest (1223 K) measurement temperatures, along with their respective activation energies. As previously discussed, the NiO secondary phase identified in the SEM‐EDXS studies has an insignificant impact on the oxygen permeation of the NNO ceramics, owing to its low concentration. At 1223 K, the oxygen flux of the *a*,*b*‐plane textured membrane is 2.17 times higher than that of the randomly oriented sample, while the oxygen permeation in the NNO ceramic with grains aligned along their *c*‐axis is about twofold lower than that of the non‐oriented counterpart. At 1023 K, the oxygen performance of the textured bulk membrane with grain orientation along the *a*,*b*‐plane is improved by a factor of 2.82 compared to the non‐textured bulk, which in turn has an oxygen transport flux ≈1.4 times greater than the *c*‐axis textured ceramic. Murata et al.^[^ ^85^ ^]^ investigated the electrical conductivity of textured NNO bulks between 473–1073 K and compared the results with those of a non‐textured sample. Due to the anisotropic nature of this oxide, the electrical conductivity in the *a*,*b*‐plane textured specimen was roughly twice as high as that in the non‐textured bulk, while the electrical conductivity of the *c*‐axis textured ceramic was approximately an order of magnitude lower than that of the randomly oriented counterpart. These results align with the oxygen transport measurements, confirming that electron and oxygen‐ion transport in NNO is substantially higher along the *a*,*b*‐plane compared to the *c*‐axis, as described in the literature.^[^ ^27^, ^29^, ^41^, ^42^, ^43^, ^44^, ^45^, ^46^, ^47^ ^]^ Table 2 also includes the results of the oxygen transport measurements from asymmetric membranes studied by Zhao et al.^[^ ^84^ ^]^ These feature dense, NNO films with grains oriented with their *a*,*b*‐plane parallel to the oxygen permeation direction, achieved through magnetic alignment on a porous support, leading to a significant enhancement in oxygen flux. Furthermore, asymmetric ceramics containing dense films with randomly oriented NNO grains were produced and their oxygen transport properties were investigated (Table 2). In the present work, this concept has been successfully extended to a bulk membrane design. When comparing the oxygen permeation values measured at 1023 and 1223 K, it is evident that the asymmetric disk membrane reported by Zhao et al.^[^ ^84^ ^]^ exhibits oxygen permeation fluxes approximately double those of the *a*,*b*‐plane textured bulk ceramic in this study. To achieve comparable oxygen permeation values, a reduction in membrane thickness seems necessary. For instance, using a 0.5 mm thick bulk membrane might result in oxygen permeation flux at 1223 K that closely aligns with the study of Zhao et al.^[^ ^84^ ^]^ Therefore, a study examining the influence of membrane thickness on oxygen permeation is essential for accurate assessment. This would help to determine the characteristic membrane thickness (*L*_c_), at which both surface oxygen exchange and bulk oxygen diffusion have an equal impact on oxygen permeation.^[^ ^132^, ^135^ ^]^ *L*_c_ is also accessible when the oxygen bulk diffusion coefficient D* and the oxygen surface exchange coefficient k* are known, as it is defined by the ratio of D* to k* (see Equation S4, Supporting Information).^[^ ^136^ ^]^ Using data from the literature on NNO single crystals and bulk ceramics, *L*_c_ was calculated at various temperatures and is summarized in Tables S4–S6 (Supporting Information).^[^ ^34^, ^35^, ^48^, ^123^, ^137^ ^]^ In almost all cases, a temperature dependence is evident, with *L*_c_ diminishing as the temperature decreases. However, the estimated *L*_c_ values vary significantly, leading to a lack of clear trends. This variability likely arises from discrepancies in experimental techniques, differences in sample quality, and fluctuations in the specific conditions under which the measurements were conducted.

**Table 2:** Summary of the oxygen permeation fluxes measured at 1023 and 1223 K for the NNO ceramic membranes in this study, including corresponding activation energies and comparative results from Zhao et al.^[^ ^84^ ^]^

Disk‐shaped membrane^a)^	Dense layer thickness [mm]	Oxygen flux $J_{O_{2}}\;$ (He sweep) [mL min^−1 ^cm^−2^]			Activation energy *E*_a_ [eV]	Oxygen flux $J_{O_{2}}\;$ (CO_2_ sweep) [mL min^−1 ^cm^−2^]	Ref.
		1023 K		1223 K		1223 K	
*a*,*b*‐plane textured NNO bulk ceramic	1.00	0.31	1.02		0.59	0.74 (140 h)	this work
randomly oriented NNO bulk ceramic	1.00	0.11	0.47		0.78	−	this work
*c*‐axis textured NNO bulk ceramic	1.00	0.08	0.23		0.64	−	this work
randomly oriented NNO bulk ceramic	1.00	0.14	0.53		0.73	−	[84]
asymmetric ceramic with *a*,*b*‐plane textured NNO film	0.162	0.74	2.03		0.55	1.40 (120 h)	[84]
asymmetric ceramic with randomly oriented NNO film	0.129	0.23	0.98		0.79	−	[84]

^a)^ The membrane thickness totals 1 mm.

Arrhenius plots (Figure  7c) were utilized to determine the activation energy *E*_a_ for oxygen permeation. The data points were analyzed using linear regression, with *E*_a_ derived from the slope of the resulting fit. The calculated *E*_a_ values are listed in Table 2, alongside those from Zhao et al.^[^ ^84^ ^]^ A direct comparison is feasible since the same measurement conditions (see Experimental Section) and oxygen permeation setup were employed. Table S7 (Supporting Information) provides an overview of the *E*_a_ values reported in the literature for NNO single crystals and polycrystalline ceramics.^[^ ^34^, ^35^, ^48^, ^138^, ^139^, ^140^ ^]^ These values were determined using methods distinct from oxygen permeation measurements. As displayed in Table 2, introducing texture into the NNO bulk membranes reduces *E*_a_. In line with the results of Zhao et al.^[^ ^84^ ^]^ for the asymmetric ceramic samples, the *a*,*b*‐plane textured membrane in this work exhibits the lowest *E*_a_ within the studied temperature range of 1023–1223 K. This results from the preferential grain orientation along this plane, which favors oxygen transport and reduces the energy required for oxygen migration. Interestingly, the *E*_a_ of the *c*‐axis textured NNO bulk ceramic, with a value of 0.64 eV, is lower than that of the polycrystalline sample (0.78 eV). This observation is consistent with findings from an earlier study by Escobar Cano et al.,^[^ ^73^ ^]^ which showed that *c‐*axis texturing of La_2_NiO_4+_ *_δ_* ceramic membranes reduced both oxygen permeation and *E*_a_. As the level of *c*‐axis texturing increased, the oxygen fluxes and *E*_a_ values decreased relative to the non‐textured membrane. The lower *E*_a_ observed for *c*‐axis textured membranes is attributed to the role of O⁻ ions in oxygen transport, as evidenced in oxygen diffusivity measurement in studies by Bassat et al.^[^ ^43^ ^]^ on La_2_NiO_4+_ *_δ_* single crystals. These O⁻ ions, present at much lower concentrations than O^2−^ ions, lead to reduced D* values along the *c*‐axis. However, their smaller size and charge contribute to higher mobility, effectively lowering the *E*_a_ for oxygen transport along this axis.^[^ ^43^, ^73^, ^141^ ^]^ Given the structural similarity between NNO and La_2_NiO_4+_ *_δ_*, the presence of O⁻ ions may likewise contribute to the reduced *E*_a_ value for oxygen permeation in the *c*‐axis textured NNO membrane compared to the polycrystalline sample. The presence of O⁻ species was confirmed through X‐ray photoelectron spectroscopy (XPS) measurements, as detailed later. The *E*_a_ value of randomly oriented bulk NNO ceramic from this work is comparable to those estimated by Zhao et al.^[^ ^84^ ^]^ but differs slightly from the literature values in Table S7 (Supporting Information) due to the variations in measurement methods and temperatures. On the other hand, the activation energies for *a*,*b*‐plane, and *c*‐axis textured NNO bulk ceramics are significantly lower than those documented in Table S7 (Supporting Information) for polycrystalline ceramics. This discrepancy can be attributed to the texturing effect. Furthermore, the CO_2_ stability of the NNO ceramic membrane with preferred grain alignment along the *a*,*b*‐plane was assessed during oxygen permeation at 1223 K. To this end, a long‐term stability measurement was performed by switching the sweep gas from He to CO_2_. As inferred from Figure  7d, the initial flux averaged 1.01 mL min⁻¹ cm⁻^2^ under He as the sweep gas. After ≈50 h, the sweep gas was switched to CO_2_, and the oxygen flux stabilized at 0.74 mL min⁻¹ cm⁻^2^ for ≈140 h. When the sweep gas was returned to He, the oxygen permeation flux recovered to a value comparable to that observed before the CO_2_ stability test (=0.99 mL min⁻¹ cm⁻^2^). When using the synthetic air/CO_2_ gradient, the oxygen flux of the *a*,*b*‐plane textured NNO ceramic at 1223 K decreased by ≈27.5%. Zhao et al.^[^ ^84^ ^]^ (see Table 2) and other researchers have reported a similar reduction during stability tests with CO_2_ sweep on OTMs.^[^ ^23^, ^142^, ^143^, ^144^ ^]^ This drop can be traced back to CO_2_ adsorption on the membrane surface, inhibiting the surface exchange reaction. The absorbed CO_2_ species react with the oxygen vacancies on the membrane surface, impeding oxygen desorption from the surface and therefore reducing the flux. Upon switching back to He, the original oxygen permeation flux was restored as CO_2_ desorbed.^[^ ^23^, ^84^, ^142^, ^143^, ^144^, ^145^ ^]^ For potential industrial applications, it is crucial that the material not only sustains a high and stable oxygen permeation flux during extended operation but also remains resistant to disintegration in various reducing environments, such as CH_4_ and CO_2_.^[^ ^142^, ^146^, ^147^ ^]^

**Figure 8:**
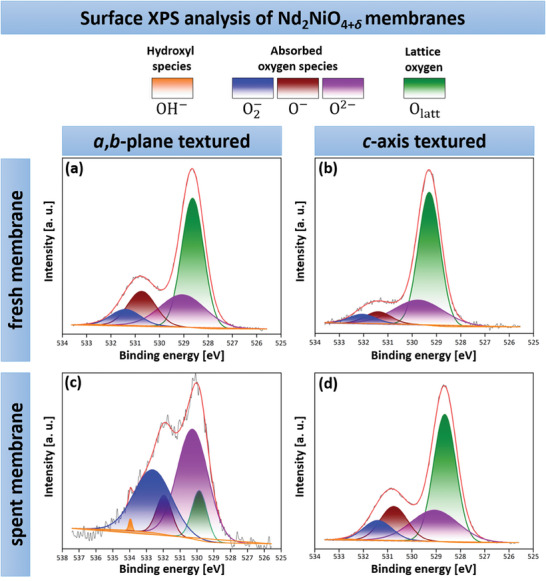
High‐resolution XPS spectra of the O 1s region, obtained from the surface (feed‐side) of both a,b) fresh and c,d) spent *a*,*b*‐plane and *c*‐axis textured NNO ceramic membranes after oxygen permeation.

To identify the oxygen species participating in the oxygen transport mechanism of NNO, as illustrated in Figure  1b,c, XPS analysis was conducted on the surface (feed side) of the textured NNO ceramics, both before (fresh membranes) and after exposure to synthetic air during the oxygen permeation measurements (spent membranes). High‐resolution O 1s core‐level XPS spectra for fresh ceramics are presented in **Figure**  **8**a,b, and for spent ceramics in Figure  8c,d. Each spectrum exhibits two distinct peaks, both highlighted in red. The first, at a lower binding energy, corresponds to lattice oxygen ions O_latt_ (green area), while the second, at a higher binding energy, is attributed to adsorbed oxygen species.^[^ ^148^, ^149^, ^150^, ^151^ ^]^ The second peak can be also assigned to various oxygen species, including O^2 −^ (purple area), O^−^ (brown area), and $O_{2}^{-}$ (blue area), which play a role in the oxygen transport mechanism and are collectively referred to as absorbed oxygen species O_ads_.^[^ ^41^, ^43^, ^148^, ^149^, ^150^, ^151^, ^152^, ^153^ ^]^ Additionally, this peak is also related to hydroxyl species OH^−^ (orange area), formed when absorbed oxygen species react with moisture.^[^ ^151^, ^154^ ^]^ The binding energies of the various oxygen species for each sample are summarized in Table S8 (Supporting Information). Due to the anisotropic properties of NNO, oxygen transport predominantly occurs along the *a*,*b*‐plane rather than the *c*‐axis. As a result, after the oxygen permeation measurements, a higher concentration of absorbed oxygen species O_ads_ is anticipated on the surface of the spent *a*,*b*‐textured ceramic compared to the *c*‐axis textured specimen. By analyzing the peak areas of each oxygen species in the O 1s XPS spectra, the O_ads_/O_latt_ ratio can be determined. Both the peak areas and the ratios are provided in Table S9 (Supporting Information). A higher O_ads_/O_latt_value indicates a greater involvement of absorbed oxygen species (O^−^, O^2 −^, and $O_{2}^{-}$) in the oxygen transport mechanism. The O_ads_/O_latt_ ratio for the fresh *a*,*b*‐plane textured NNO ceramic was 1.01, which increased significantly to 8.53 after the oxygen permeation. This substantial improvement is attributed to the favorable grain orientation along the *a*,*b*‐plane, which facilitates enhanced oxygen transport and allows for better adsorption of oxygen species on the membrane surface, as observed in the O 1s XPS spectra (see Figure  8a,c). In contrast, for the fresh *c*‐axis textured sample, the O_ads_/O_latt_ ratio was 0.62, and it elevated slightly to 1.03 after the permeation test. This indicates moderate oxygen adsorption, which is significantly lower than the *a*,*b*‐plane textured membrane. The restricted oxygen transport along the *c*‐axis, due to the less favorable grain orientation, results in fewer absorbed oxygen species, as reflected in the O 1s XPS spectra (see Figure  8b,d). The XPS results highlight the anisotropic oxygen transport behavior, with the *a*,*b*‐plane textured membrane exhibiting significantly higher adsorption of oxygen species compared to the *c*‐axis textured ceramic. This is due to the more favorable grain orientation along the *a*,*b*‐plane, which enhances oxygen diffusion and adsorption, thereby improving oxygen transport efficiency. These findings align with the oxygen permeation results shown in Figure  7.

**Figure 9:**
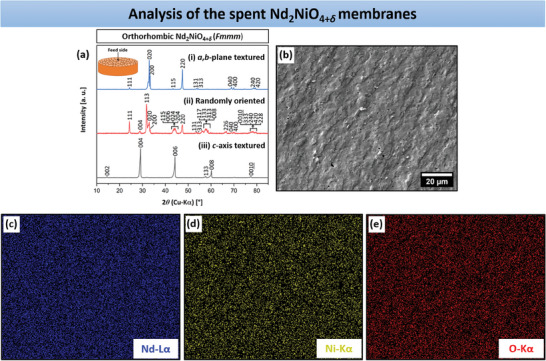
a) XRD patterns recorded from the feed side of the spent NNO bulk membranes. b) SEM micrograph showing the fractured cross‐section of the spent *a*,*b*‐plane textured NNO ceramic after oxygen permeation, and c–e) respective individual EDXS element maps of Nd (blue), Ni (yellow), and O (red).

After the oxygen permeation measurements, the three spent NNO bulk membranes were subjected to XRD and SEM‐EDXS analysis to assess their phase structure and morphology. **Figure**  **9**a shows XRD patterns from the membrane feed sides, confirming the robust stability of the NNO material. The absence of secondary phases and the exclusive detection of orthorhombic NNO rule out any material degradation. NiO thus persists only as a trace impurity, undetectable by XRD, and has no impact on NNO's integrity. The fractured membrane cross‐sections were then characterized using SEM‐EDXS. The SEM micrograph of the fractured cross‐section of the *a*,*b*‐plane textured NNO membrane (Figure  9b) reveals no significant morphological changes after exposure to He and CO_2_ as sweep gases during oxygen permeation. In the EDXS element maps (Figure  9c–e), no foreign phases were found, as Nd, Ni, and O were uniformly distributed across the sample area. The post‐characterization of the other two spent NNO membrane materials by SEM‐EDXS, illustrated in Figures S10 and S11 (Supporting Information), also disclosed no structural changes or material degradation. The dense bulk NNO membrane with grains oriented in the *a*,*b*‐plane thus emerges as an excellent candidate for potential industrial applications, demonstrating both substantial long‐term chemical stability and high oxygen permeation performance. When comparing the oxygen transport properties of the *a*,*b*‐plane textured NNO disk with those of other disk‐shaped bulk membranes of K_2_NiF_4_, perovskite, or dual‐phase types (as outlined in Table S10, Supporting Information), the NNO membrane stands out due to its superior characteristics previously discussed.^[^ ^7^, ^23^, ^84^, ^129^, ^142^, ^155^, ^156^, ^157^, ^158^, ^159^, ^160^, ^161^ ^]^ Although perovskite‐based OTMs exhibit the highest oxygen permeation flux under He sweeping conditions, their performance deteriorates rapidly in a CO_2_ atmosphere due to the formation of carbonates on the membrane surface, which limits their commercial viability.^[^ ^3^, ^13^, ^18^, ^19^, ^20^, ^21^, ^22^ ^]^ The NNO bulk ceramic with oriented grains along the *a*,*b*‐plane using magnetic fields, shows an oxygen permeation flux of 0.74 mL min⁻¹ cm⁻^2^ under CO_2_ sweep conditions. This value is approaching the commercial threshold of 1.00 mL min⁻¹ cm⁻^2^, suggesting that the NNO membrane is nearing feasibility for potential industrial applications.^[^ ^146^, ^147^ ^]^

## 3 Conclusions

This study has demonstrated the effective production of textured Nd_2_NiO_4+_ *_δ_* bulk membranes by orienting grains in a 0.9 T magnetic field. The texturing extent and the microstructure of the ceramic membranes were assessed qualitatively and quantitatively using various analytical techniques. Although NiO impurities were detected, their low concentration has no effect on the oxygen permeation of the ceramic membranes. The dense Nd_2_NiO_4+_ *_δ_* disk membrane with grains aligned within the *a*,*b*‐plane displayed a high oxygen permeation flux of ≈1.02 mL min⁻¹ cm⁻^2^ under helium sweeping and superior CO₂ stability, maintaining a stable flux of ≈0.74 mL min⁻¹ cm⁻^2^ at 1223 K for at least 140 h. These attributes establish Nd_2_NiO_4+_ *_δ_* as a leading candidate for commercial applications in air separation and CO₂ capture and storage, especially when compared to CO₂‐sensitive perovskite oxygen permeation membranes.

Future research should focus on analyzing other critical factors affecting oxygen permeation in textured Nd_2_NiO_4+_ *_δ_*‐based ceramic systems. In particular, a thorough investigation into the impact of membrane thickness on oxygen permeation is necessary. It is anticipated that oxygen permeation performance will improve with decreasing membrane thickness until reaching the characteristic membrane thickness, Furthermore, measuring the coefficients for oxygen bulk diffusion and oxygen surface exchange at various temperatures could provide deeper insights into oxygen transport behavior in Nd_2_NiO_4+_ *_δ_*. By analyzing the ratio of these coefficients, the characteristic membrane thickness can be estimated. Additionally, it would be interesting to investigate how the oxygen transport properties of textured Nd_2_NiO_4+_ *_δ_* ​ ceramic membranes respond to varying proportions of oxygen and nitrogen in synthetic air. Besides, it is important to examine how grain size, as well as the substitution or doping of Nd and/or Ni, influences oxygen permeation performance. Reviewing and comparing the effects of various sintering techniques, such as pressureless sintering, hot pressing, spark plasma sintering, and spark plasma texturing, on ceramic densification is also crucial. Each of these parameters can significantly alter the material's microstructure and transport properties, and a thorough investigation of their impact on oxygen permeability is critical. By clarifying the interplay between these factors, valuable insights can be gained, potentially leading to optimized oxygen transport performance and enhanced permeation efficiency.

## 4 Experimental Section

### Preparation of Nd_2_NiO_4+δ_ Ceramic Membranes

The NNO starting powder was synthesized via a solid‐state reaction route. For this, Nd_2_O_3_ (>99.9%, FUJIFILM Wako Pure Chemical Co., Japan) and NiO (>99.9%, FUJIFILM Wako Pure Chemical Co., Japan) were mixed in a 1:1 molar ratio, together with a small amount of ethanol, in a mortar. The powder mixture was then calcined in air at 1523 K for 12 h. After the calcination step, the powder was thoroughly ground and the resulting lump was pulverized into a fine powder using a ball mill with 4 mm zirconia balls at room temperature for 48 h. Next, the synthesized powder was dispersed in a mixed solution of 50 wt.% distilled water and 50 wt.% ethanol. Polyethyleneimine with an average molecular weight of 10.000 (FUJIFILM Wako Pure Chemical Co., Japan) was added as the dispersant to form a suspension with 46 wt.% NNO powder. Afterward, the suspension was ultrasonicated for 10 min and stirred using a magnetic stirrer to ensure proper dispersion of the NNO powder. Prior to slip casting, the suspension was degassed in a vacuum. The NNO ceramic membranes were fabricated by slip casting in a 0.9 T magnetic field. A schematic representation of the membrane production process is presented in Figure  1d. The suspension, containing NNO powder, was poured into a bottomless cylindrical vessel placed on either a porous alumina mold or an Omnipore polytetrafluoroethylene (PTFE) membrane (Merck, Japan). The porous alumina mold, fabricated by a conventional sintering manner, had a porosity of ≈40%. The Omnipore PTFE membrane featured a thickness of 65 µm, a diameter of 25 mm, a pore size of 0.45 µm, and a porosity of 80%. The alumina mold was utilized for experiments conducted without a magnetic field, while the PTFE membrane was employed when a magnetic field was applied during slip‐casting. A 0.9 T magnetic field, generated by neodymium magnets was applied during the slip casting experiment. To achieve NNO particle orientation in the *a*,*b*‐plane, the magnetic field was applied perpendicular to the casting direction. Conversely, to align the particles along the *c*‐axis, the magnetic field was applied parallel to the slip casting direction. Additionally, a slip casting experiment was conducted without using a magnetic field, leading to a random alignment of the NNO particles. The as‐prepared green compacts were debinded at 693 K for 1 h in air to remove the dispersant, then cold isostatic pressed (180 MPa, 3 min) and sintered at 1523 K for 12 h. This process resulted in dense, disk‐shaped bulk NNO ceramics with diameters between 10 and 13 mm as well as thicknesses ranging from 1.0 to 1.5 mm.

### Analytical Methods for Microstructure and Texture

Phase identification of the starting powder and ceramic membranes was conducted by X‐ray diffraction (XRD, Bruker D8 Advance, Bruker AXS GmbH, Germany) using Cu‐K*α* radiation at 40 kV and 40 mA. The XRD patterns were recorded over a 2*θ* range of 10 to 85°, with a step size of 0.01° and an acquisition time of 1s per step. The evaluation of XRD data was carried out with the subsequent powder diffraction file (PDF) from the International Centre for Diffraction Data (ICDD): Nd_2_NiO_4.20_ (PDF no. 01‐089‐0131, orthorhombic, *a* = 537.4 pm, *b* = 545.8 pm*, c* = 1238.7 pm). The Lotgering orientation factor *f*, which provided a quantitative measure of the texturing degree in the textured NNO ceramic membranes, was calculated from the collected XRD patterns applying Equation (2):^[^ ^92^, ^93^ ^]^

(2) $$f_{\mathrm{hk}0\;\mathrm{or}\;00l}=\frac{P_{\mathrm{hk}0\;\mathrm{or}\;00l}-P_{0}}{1-P_{0}}$$

where,

(3) $$P_{\mathrm{hk}0\;\mathrm{or}\;00l}=\frac{\sum I_{\mathrm{hk}0\;\mathrm{or}\;00l}}{\sum I_{\mathrm{hkl}}}$$

and

(4) $$P_{0}=\frac{\sum I_{\;\mathrm{hk}0\;\mathrm{or}\;00l}^{\;0}}{\sum I_{\;\mathrm{hkl}}^{\;0}}$$

here, *P*_*hk*0 or 00*l*_ represents the ratio of the sum of the relative intensities of all *hk*0 or 00*l* reflections (=∑*I*_*hk*0 or 00*l*_) to the sum of the relative intensities of all *hkl* reflections (=  ∑*I*_*hkl*_) for the textured NNO membrane. *P*_0_ is expressed as the ratio of the sum of the relative intensities of all *hk*0 or 00*l* reflections (= $\sum I_{\;\mathrm{hk}0\;\mathrm{or}\;00l}^{\;0}$) to the summation of the relative intensities of all *hkl* reflections (=$\sum I_{\;\mathrm{hkl}}^{\;0}$) for the non‐textured NNO sample (=initial powder). The Lotgering factor *f* was determined for the 2*θ* range from 10 to 85°.

Moreover, the texturing degree in the as‐sintered NNO membranes was investigated by measuring XRD pole figures of the (101), (004), and (220) lattice planes (tilt angle *φ*  = 0–70°, in 5° steps, azimuth angle *ψ* = 0–360°, in 5° steps, integrating *β*‐measurement 10 s·5°^−1^). The pole figures were recorded using a 5‐circle X‐ray diffractometer (3003 ETA, Seifert Analytical X‐Ray, Germany) equipped with Co‐K*α* radiation (spot focus), an X‐ray tube power of 30 kV and 40 mA, a 2 mm point collimator, and a silicon drift detector (AXAS‐M, Ketek GmbH, Germany). The pole figure data were analyzed using the LaboTex software (LaboSoft, Poland).

The oxygen content of the ceramic samples was calculated by carbothermal fusion using a hot gas analyzer (ELTRA ELEMENTRAC ONH‐p2, ELTRA GmbH, Germany). Per measurement ≈5 mg sample was placed in a nickel capsule. The graphite crucible was then loaded with ≈50 mg of graphite powder. Calibration was performed using four standards: a steel standard with 33 ± 3 ppm O, a copper standard with 251 ± 3 ppm O, a titanium standard with 1000 ± 60 ppm O, and ZrO_2_ (>99%, Sigma‐Aldrich, Germany) with 25.97 ± 0.44 wt.% O.

The microstructural and textural characterization of the NNO ceramic membranes was carried out utilizing SEM and related analytical techniques. To obtain smooth sample surfaces, vibratory‐polished cross‐sections were prepared. For this, the sintered ceramic disks were sliced into thin sections using a precision vertical diamond wire saw (Model 3242, O'Well, Germany). These slices were embedded in epoxy resin and polished with a MultiPrepTM precision polishing system (Allied High Tech Products, Inc., USA), employing diamond lapping films of varying fineness (30–3 µm). This was followed by vibratory polishing with a 50 nm alumina suspension using a VibroMet 2 vibratory polisher (Buehler, Germany). Finally, the cross‐sections were coated with a thin conductive carbon layer through thread evaporation (Leica EM SCD500, Leica Microsystems, Germany). Both the vibratory‐polished and fractured sample cross‐sections were examined for their microstructural morphology using field‐emission scanning electron microscopy (FE‐SEM, JSM‐6700F, JEOL, Japan). The secondary electron detection was performed at 2 kV, while the backscattered electrons were detected at 20 kV. The FE‐SEM, equipped with an EDXS (Oxford Instruments INCA‐300, UK) was operated at 15 kV to evaluate the elemental composition and distribution within the samples. A more in‐depth microstructural characterization was performed with another field‐emission scanning electron microscope (FE‐SEM, JSM‐7610FPlus, JEOL, Japan) coupled with dual EDXS (XFlash 6|60, Bruker, USA). SEM micrographs with backscattered‐electron channeling contrast and EDXS elemental maps were acquired at 15 kV. The grain size in the various specimens was estimated by analyzing ≈100 grains from the SEM micrographs by using ImageJ (version 1.53t).^[^ ^108^ ^]^

The density of the ceramic membranes was calculated using the Archimedes method (ISO 5018:1983) with 2‐propanol as the fluid. The theoretical density of orthorhombic NNO (space group: *Fmmm*), according to the PDF card Nd_2_NiO_4.20_ (PDF no. 01‐089‐0131), was 7.55 g cm^−3^.

The distribution of grain orientation (texture degree) in the NNO ceramic materials was analyzed using the electron backscatter diffraction (EBSD) technique with an FE‐SEM (ZEISS Sigma 300, ZEISS, Germany) operating at 20 kV and 20 nA and equipped with an EBSD detector (C‐Nano+, Oxford Instruments, UK). The specimens were mounted on an inclined sample holder with a 70° tilt relative to the electron beam, at a working distance of 12 mm. Measurements were conducted on the vibratory‐polished cross‐sections of the samples, with a step size of 180 nm step^−1^. The EBSD data were acquired, processed, and analyzed using AZtecHKL software (Oxford Instruments, UK). For the EBSD data evaluation, the crystallographic data of Nd_2_NiO_4.23_ in the orthorhombic structure (space group: *Fmmm*) from the PAULING FILE Multinaries Edition – 2022 database (Springer Materials, dataset ID: sd_1 143 456) were employed. The unit cell parameters are *a* = 537.1 pm, *b* = 545.6 pm, and *c* = 1236.5 pm.

The *a*,*b*‐plane textured NNO membrane was further examined by a field‐emission TEM (FE‐TEM, JEOL JEM2100F‐UHR, Japan) operating at 200 kV (objective lens coefficient of spherical aberration *C*_S _= 0.5 mm and coefficient of chromatic aberration *C*_C_ = 1.1 mm). For plan‐view TEM analysis, the ceramic was cut into a block measuring 1 × 1 × 2 mm, embedded in epoxy resin, and then polished to a thickness of 0.015 mm using the MultiPrep precision polishing system (Allied High Tech Products, Inc., USA) with diamond lapping films. The specimen was subsequently placed on a copper TEM grid and rendered electron‐transparent through argon ion sputtering at 3 kV with an incidence angle of 6°, using a Precision Ion Polishing System (PIPS, model 691, Gatan, Inc., USA). The FEM‐TEM was fitted with an EDXS (Oxford Instruments INCA 200, UK) as well as a Gatan energy filter (GIF 2001) with a 1 k charge‐coupled device camera. The microscope was utilized in various modes: bright‐field (BF), dark‐field (DF), HRTEM, and STEM in high‐angle annular dark field (HAAD) imaging. Additionally, SAED was performed.

Multislice simulation was made using jems – Electron Microscopy Software Java Version (version 5.2031u2024b11, P. Stadelmann, JEMS‐ Swiss, Lausanne, Switzerland). The diameter of the objective aperture was set to 36 mrad, and at a defocus of 42 nm a sample thickness of 45.96 nm (i.e., after iterated 60 slices) was selected. The crystal structure was read from the Inorganic Crystal Structure Database ICSD no. 50 440 (ICSD release 2024.1, FIZ Karlsruhe GmbH, Karlsruhe, Germany,), which corresponds to PDF no. 01‐089‐0131. SAED patterns were analyzed with DigitalMicrograph (version 1.70.16, Gatan Inc., USA) and CaRIne Crystallography (version 3.1, Uteam‐Divergent, France). The crystal structure of orthorhombic NNO in the *Fmmm* space group was modeled with VESTA^[^ ^162^ ^]^ (version 3.5.8, JP‐Minerals, Japan) and referenced using the PAULING FILE Multinaries Edition – 2022 database (Springer Materials, dataset ID: sd_1143456).

XPS was performed on the surface of the disk‐shaped textured NNO membranes, both before and after oxygen permeation measurements, to identify and analyze the oxygen species involved in oxygen transport mechanisms. The studies were conducted using a PHI VersaProbe III (Physical Electronics GmbH, Feldkirchen, Germany) XPS system. Micro‐focused Al‐K*α* X‐rays (1486.7 eV, 50 W, 15 kV) with a spot size of 200 µm were utilized as the X‐ray source. The surface oxygen species in the NNO materials were identified by recording high‐resolution spectra of the O 1s peaks, using a pass energy of 27 eV, a step size of −0.05 eV, and a data acquisition time of −2.4 s per point. Before the XPS measurements, the samples were sputtered with Ar ions for 1 min using a beam energy of 2 kV and a sputter area of 2 × 2 mm^2^. Charge correction of the ceramic specimens was carried out using the C 1s peak corresponding to C─C bonding at 284.8 eV. The raw XPS data were processed and analyzed with MultiPak software (version 9.8.0.19, ULVAC‐PHI, Inc., Japan), utilizing Gaussian curve fitting and Shirley baseline correction.

### Characterization of Oxygen Transport Properties

Oxygen permeation measurements on the NNO bulk membranes were conducted over a temperature range of 1023–1223 K in 50 K steps using a home‐made high‐temperature permeation cell, as detailed in previous studies.^[^ ^163^, ^164^, ^165^, ^166^ ^]^ The disks were polished to a thickness of 1 mm using 200‐grit SiC sandpaper (CarbiMet, Buehler, Germany) and then sealed onto an alumina tube (inner diameter: 10 mm) with a commercial gold conducting paste (C 5754 B, Heraeus, Germany), which was cured at 820 K for 2 h. Both the sealing process and oxygen permeation measurements were performed in a vertical tubular furnace, with heating and cooling rates set at 1 K min⁻¹. Once the target temperature was reached, it was held constant for 2 h. Synthetic air (80 vol% N_2_ and 20 vol% O_2_) was then supplied to the membrane feed side at a flow rate of 150 ml min^−1^, while helium (≥99.995%, Linde, Germany) or CO_2_ (≥99.995%, Linde, Germany) was introduced to the sweep side at a flow rate of 29 ml min^−1^. The gas flow rates were regulated using mass flow controllers (EL‐Flow, Bronkhorst, The Netherlands). The effluent composition was subsequently analyzed every 7 min with an online gas chromatograph (Agilent 7890A, Agilent, Germany) equipped with a Carboxen 1000 column (Merck, Germany), providing ≈17 data points at each temperature. The total effluent flow rate was calculated employing neon as a reference gas (≥99.995%, Linde, Germany) at a flow rate of 1 ml min^−1^. The relative leakage of O_2_ due to insufficient sealing was estimated by measuring the permeated N_2_ concentration and subtracting this value from the O_2_ permeation flux calculation. Under the assumption, that the leakage of O_2_ and N_2_ obeys the Knudsen diffusion, the fluxes of leaked N_2_ (= $J_{N_{2}}^{\mathrm{leak}}$) and O_2_ (= $J_{O_{2}}^{\mathrm{leak}}$) were correlated by Equation (5).

(5) $$J_{N_{2}}^{\mathrm{leak}}\;:\;J_{O_{2}}^{\mathrm{leak}}=\sqrt{\frac{32}{28}}\;\;x\;\frac{80\%}{20\%}=4.28$$

whereby 32 and 28 are the molar masses of N_2_ and O_2,_ respectively, and 80% and 20% correspond to the percentage of N_2_ and O_2_ in the synthetic air, respectively. Considering Equation (5), the oxygen permeation flux *J*(O_2_) through the membranes could be estimated by using Equation (6).

(6) $$J\left(O_{2}\right)=\left[c\left(O_{2}\right)-\frac{c(N_{2})}{4.28}\right]\;x\frac{F}{S}$$

here, *c*(O_2_) and *c*(N_2_) are the measured oxygen and nitrogen concentrations on the membrane sweep side, *F* represents the total flow rate of the sweep stream, and *S* is the effective permeated membrane area.^[^ ^25^, ^73^, ^165^, ^166^, ^167^ ^]^

## Conflict of Interest

The authors declare no conflict of interest.

## Supporting information



Supporting Information

## Data Availability

The data that support the findings of this study are available from the corresponding author upon reasonable request.
