# Supplementary material for: Tailoring the Anisotropic Oxygen Transport Properties in Bulk Ceramic Membranes Based on a Ruddlesden–Popper Oxide by Applying Magnetic Fields
Source: Adv Sci (Weinh). 2025 Feb 3;12(12):2411251. doi: 10.1002/advs.202411251 (PMC11948041; doi:10.1002/advs.202411251)
Supplement: Supplementary file 1 — Supporting Information [file ADVS-12-2411251-s001.docx]

Supporting Information

Tailoring the Anisotropic Oxygen Transport Properties in Bulk Ceramic Membranes Based on a Ruddlesden−Popper Oxide by Applying Magnetic Fields

Giamper Escobar Cano*, Motohide Matsuda, Zhijun Zhao, Frank Steinbach, Bernd Breidenstein, Hilke Petersen, Andreas Graff, Marc Widenmeyer, Anke Weidenkaff, Armin Feldhoff*

G. Escobar Cano, Z. Zhao, F. Steinbach, A. Feldhoff

Institute of Physical Chemistry and Electrochemistry

Leibniz University Hannover

Callinstraße 3A, D-30167 Hannover, Germany
E-mail: giamper.escobar@pci.uni-hannover.de, armin.feldhoff@pci.uni-hannover.de

M. Matsuda
Faculty of Advanced Science and Technology

Kumamoto University

2-39-1 Kurokami, Chuo-ku, 860-8555 Kumamoto, Japan

B. Breidenstein, H. Petersen

Institute of Production Engineering and Machine Tools

Leibniz University Hannover

An der Universität 2, D-30823 Garbsen, Germany

A. Graff

Fraunhofer Institute for Microstructure of Materials and Systems IMWS

Walter-Hülse-Straße 1, D-06120 Halle, Germany

M. Widenmeyer, A. Weidenkaff

Department of Materials and Earth Sciences

Technical University of Darmstadt

Peter-Grünberg-Straße 2, D-64287 Darmstadt, Germany


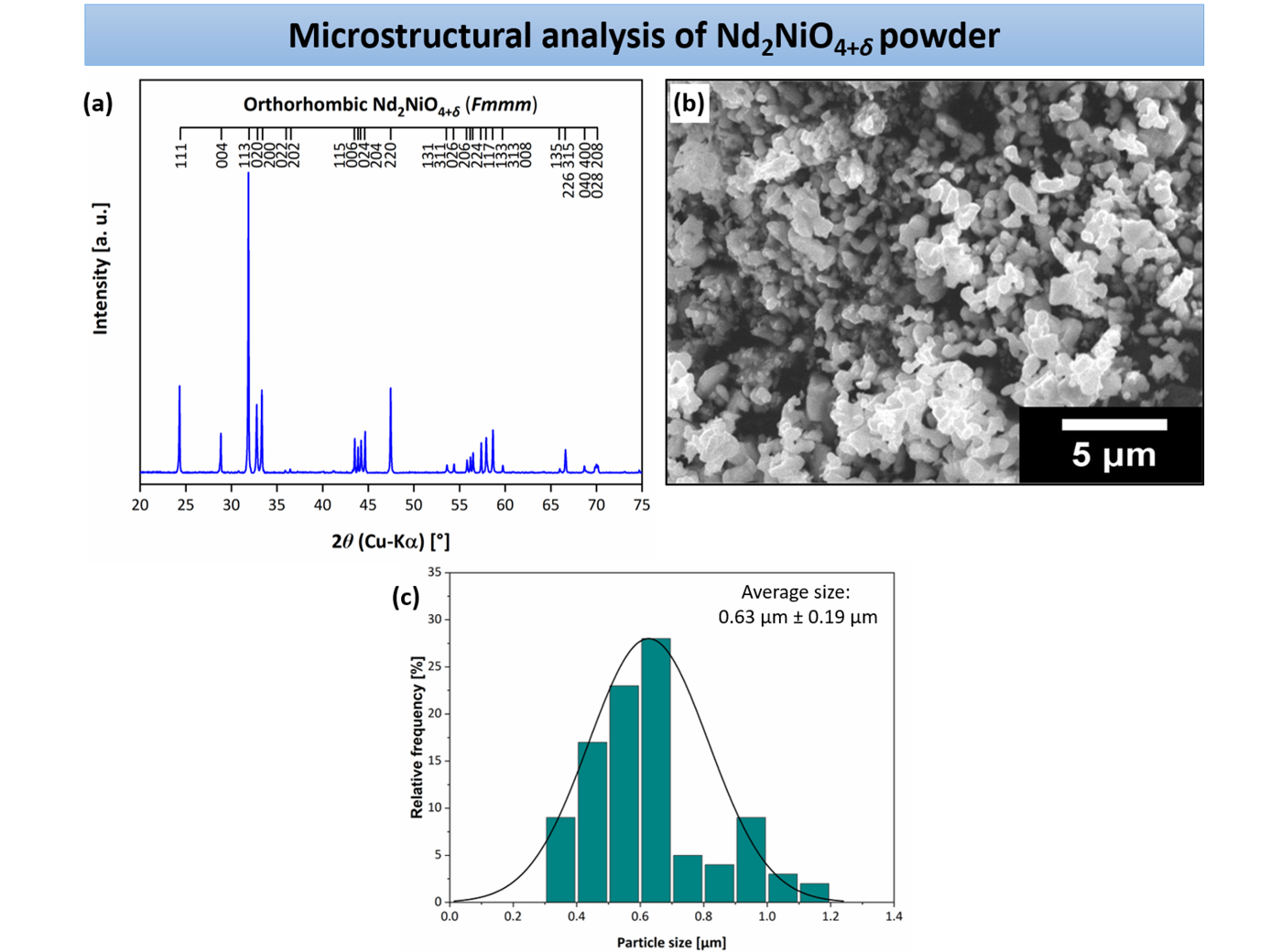


**Figure S1.** a) XRD pattern and b) SEM micrograph of the NNO powder used in the fabrication of textured bulk membranes via magnetic alignment. c) Particle size distribution of the powder, fitted with a log-normal distribution density function.

**Calculation of the magnetic anisotropy energy**

The magnetic anisotropy energy ($\text{Δ}\text{E}$) was determined for the *a*,*b*-plane-textured, *c*-axis-textured, and randomly oriented NNO ceramic membranes using **Equation 1**:^[1–3]^

$\text{∆}\text{E }\text{=}\frac{\text{∆}\text{χ}V_{\text{grain}}\text{B}^{\text{2}}}{\text{2}\text{μ}_{\text{0}}}$ (1)

where $\text{∆}\text{χ}\text{ =} \text{χ}_{\text{a}\text{,}\text{b}}\text{-}\text{ }\text{χ}_{\text{c}}\text{,}\text{ }$representing the difference in magnetic susceptibility between the *a*,*b*-plane ($\text{χ}_{\text{a}\text{,}\text{b}}$) and the *c*-axis ($\text{χ}_{\text{c}}$) of NNO. For the randomly oriented specimen, $\text{∆}\text{χ}\text{ }$is used, while for the textured ceramics, either $\text{χ}_{\text{a}\text{,}\text{b}}$ or $\text{χ}_{\text{c}}$ is applied. The susceptibility values ($\text{χ}$) can be obtained from the molar magnetic susceptibility ($\chi^{m})$ data reported by Maity et al.^[4]^ using **Equation S1**. Their magnetic susceptibility measurements were conducted along both the *a*,*b*-plane and the *c*-axis on an NNO single crystal with a superconducting quantum interference device magnetometer in the zero-field-cooled configuration, under a magnetic field of 1 T.

$\chi=\frac{\chi^{m}}{M \cdot\rho}$ (S1)

Here, *M* is the molar mass of NNO, which is 411.17 g mol^−1^, and $\text{ρ}$ is the experimental density of each NNO sample from **Table 1**.

In **Equation 1**, $\text{V}_{\text{grain}}$ designates the volume of each NNO grain. Assuming that the grains are nearly spherical, the grain volume was determined using $V_{\mathrm{sphere}}= \frac{\pi d^{3}}{6}$, where *d* denotes the grain diameter, as listed in **Table 1**. *B* is the applied magnetic field of 0.9 T, and $\text{μ}_{\text{0}}$ is the vacuum permeability, which is 1.26·10^−6^ m T A^−1^.^[5]^ The data required for calculating $\text{Δ}\text{E}$ for all three NNO samples are summarized in **Table S1**.

**Table S1.** Calculated magnetic anisotropy energies of various NNO ceramic samples used in this work.

| NNO  ceramic membrane | Molar magnetic susceptibility $\chi^{m}$[cm^3^ mol⁻^1^]^[4]^ | Magnetic susceptibility $\text{χ}$ | Grain volume  $\text{V}_{\text{grain}}$ [m^3^] | Magnetic anisotropy energy $\text{Δ}\text{E}$ [J] |
| --- | --- | --- | --- | --- |
| *a*,*b*-plane textured | $\text{χ}_{a,b}^{m}$ = 2.42·10⁻^3^ | $\text{χ}_{\text{a}\text{,}\text{b}}$= 4.37·10⁻^5^ | 9.80·10⁻^17^ | 13.80·10⁻^16^ |
| *c*-axis  textured | $\text{χ}_{c}^{m}$ = 0.86·10⁻^3^ | $\text{χ}_{c}$ = 1.55·10⁻^5^ | 6.01·10⁻^17^ | 2.99·10⁻^16^ |
| randomly oriented | ${\Delta\text{χ}}^{m}$ = 1.56·10⁻^3^ | $\text{χ}$ = 2.83·10⁻^5^ | 4.58·10⁻^17^ | 4.17·10⁻^16^ |


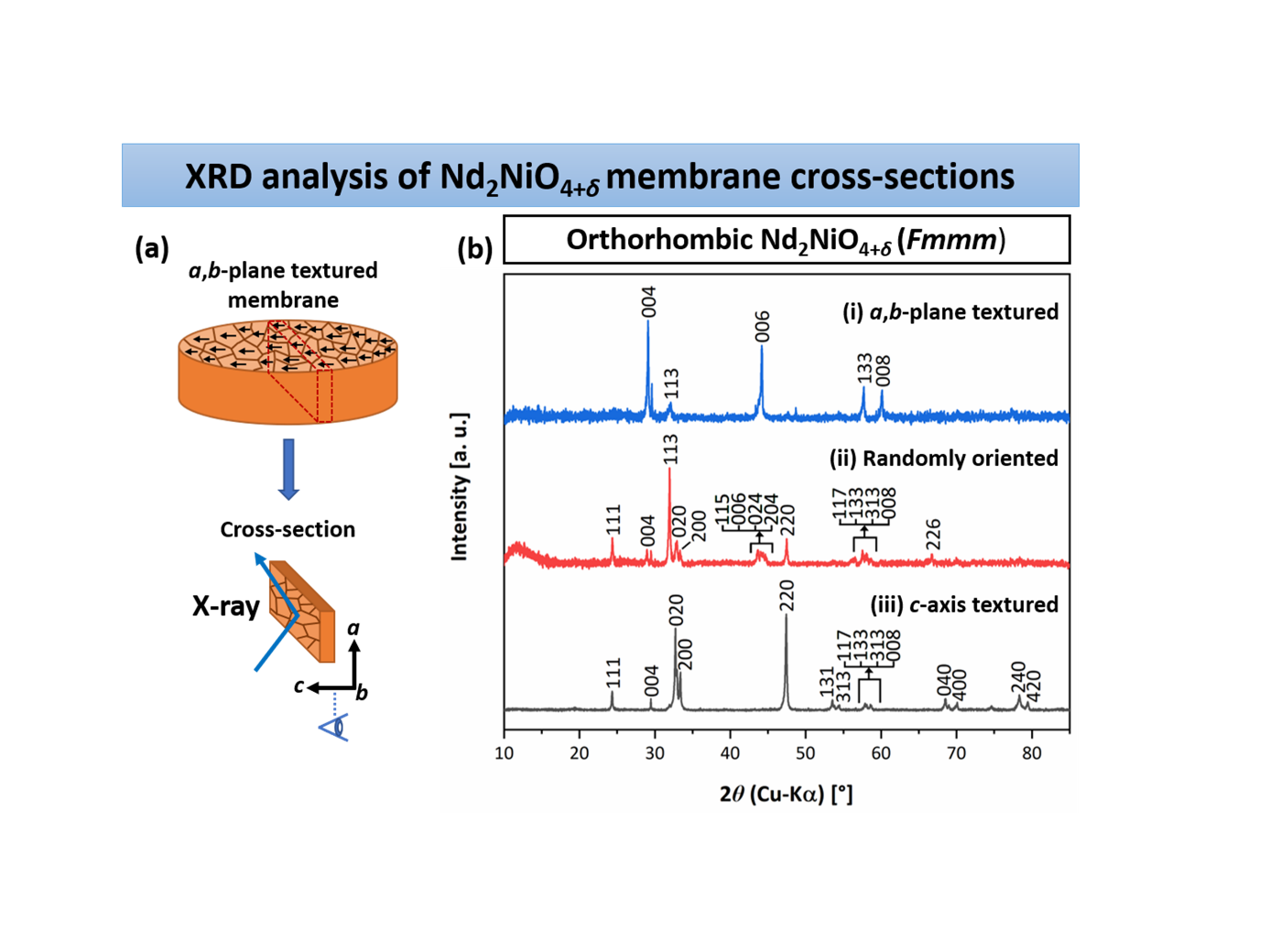


**Figure S2.** a) Sketch of XRD measurement on the cross-section of the *a*,*b*-plane textured NNO membrane, where the grains are aligned with their *c*-axis relative to the X-ray beam. b) XRD patterns recorded from the cross-sections of the (i) *a*,*b*-plane textured, (ii) randomly oriented, and (iii) *c*-axis textured bulk membranes.


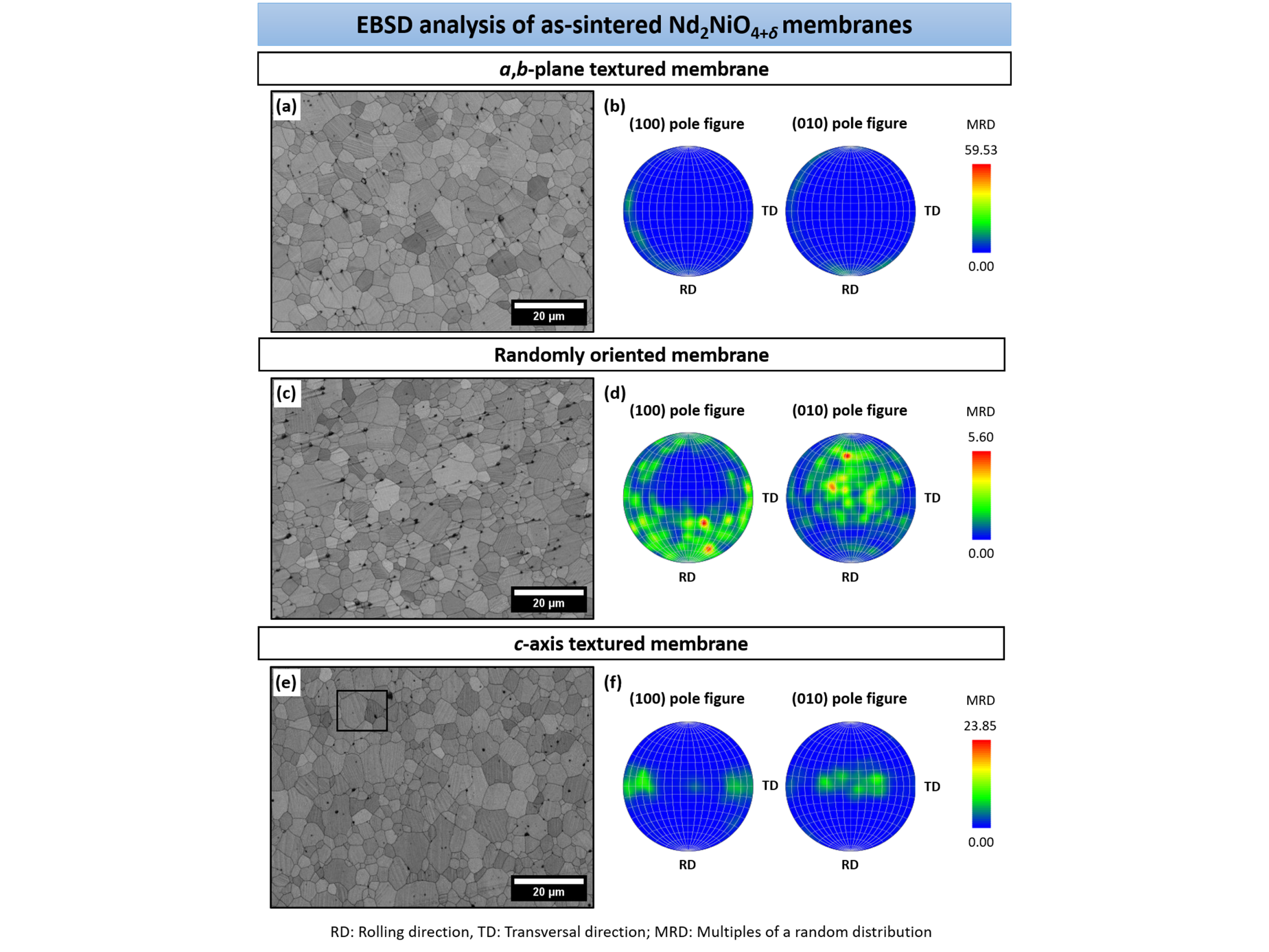


**Figure S3.** EBSD band contrast images along with (100) and (010) pole figures for the cross-section of a,b) *a*,*b*-plane textured, c,d) randomly oriented, and e,f) *c*-axis textured NNO ceramics. Different intensity scales are used for the MRD values in each case. The marked area in f) depicts two grains having different orientations, while the dark spots in the band contrast images suggest the presence of NiO, which was subsequently confirmed by SEM-EDXS analysis.


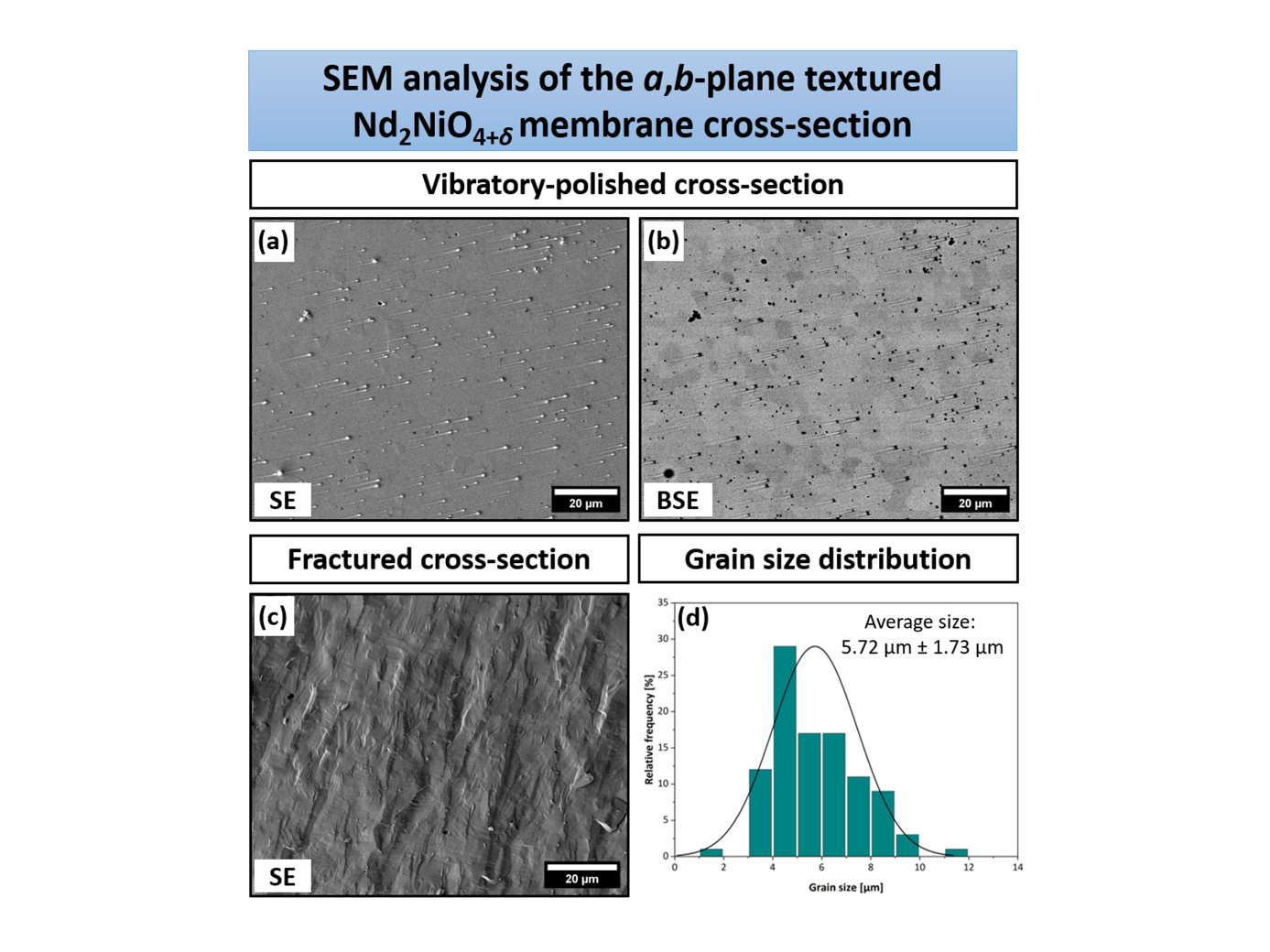


**Figure S4.** a) SEM secondary electron (SE) and b) backscattered electron (BSE) micrographs of the vibratory-polished cross-section, as well as c) SEM-SE image of the fractured cross-section obtained from the *a*,*b*-plane textured NNO bulk ceramic. d) Ceramic grain size distribution, modeled with a log-normal distribution density function.


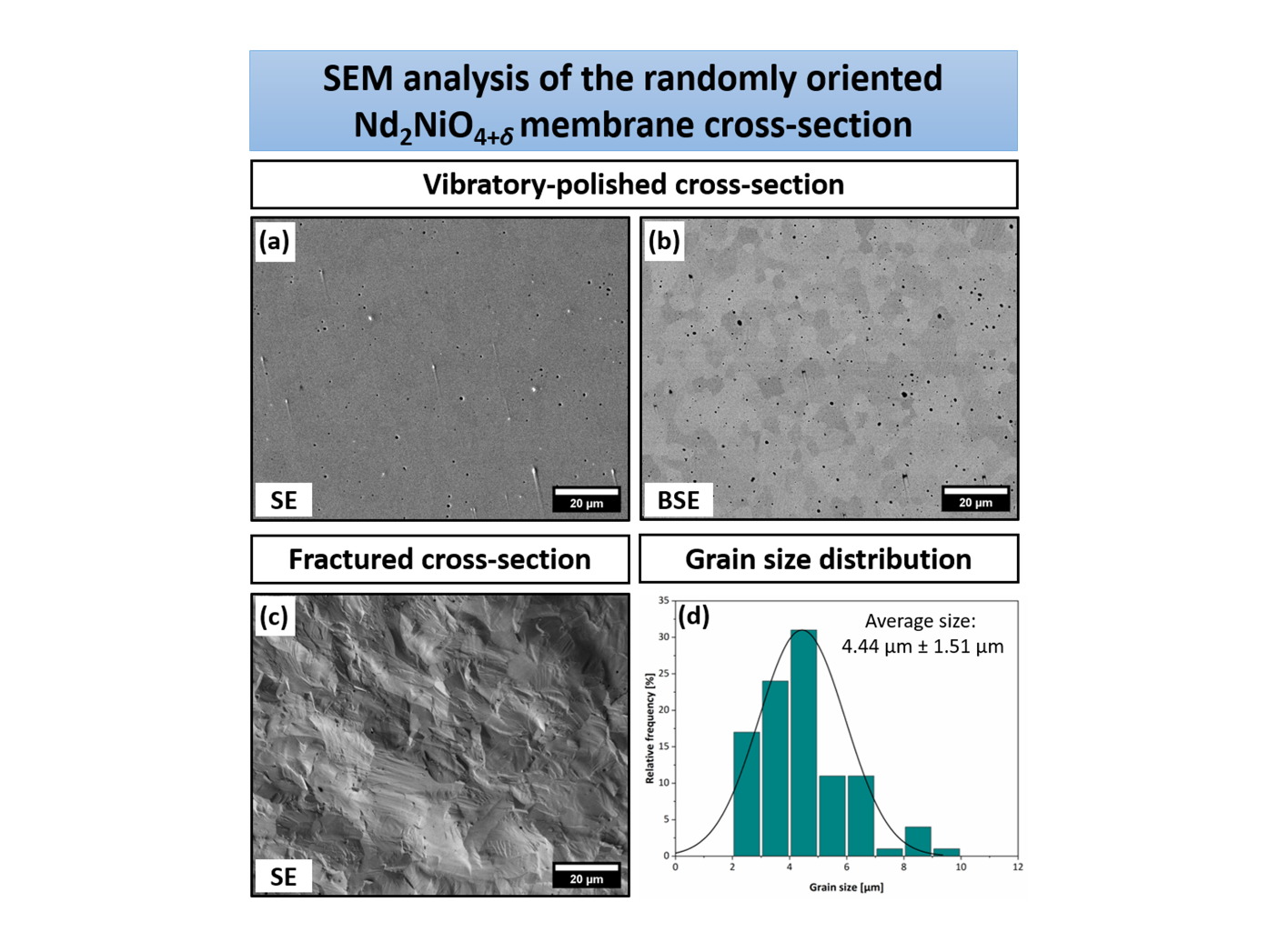


**Figure S5.** a) SEM-SE and b) SEM-BSE micrographs of the vibratory-polished cross-section, and c) SEM-SE image of the fractured cross-section from the NNO membrane with random grain orientation. d) Grain size distribution presented as a histogram and fitted with a log-normal distribution density function.


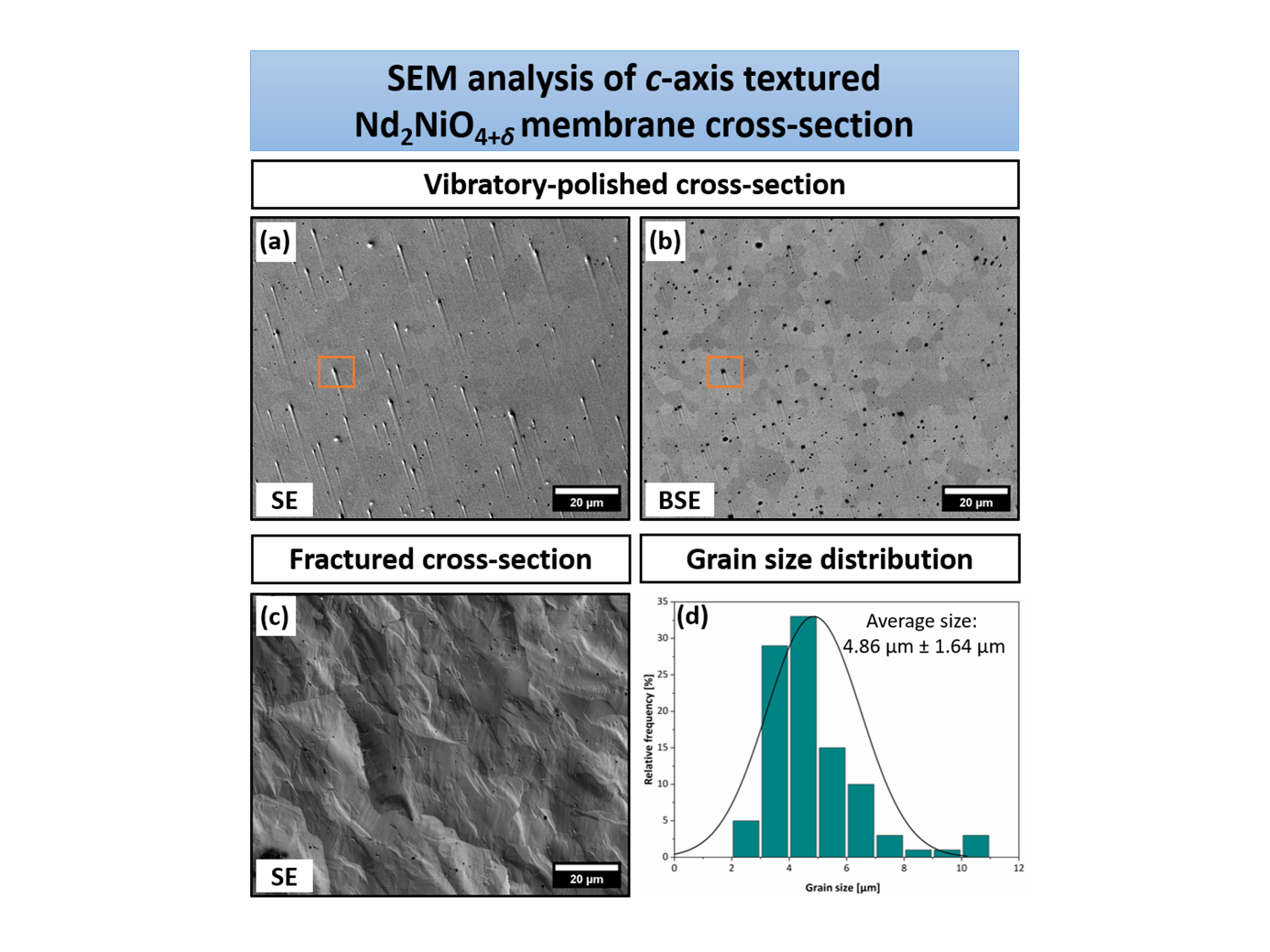


**Figure S6.** a) SEM-SE and b) SEM-BSE micrographs of the cross-section prepared by vibration polishing, and c) SEM-SE micrograph of the crushed cross-section of the *c*-axis textured NNO ceramic. d) Histogram of grain size distribution, fitted with a log-normal distribution density function. The insets in a,b) highlight the presence of some NiO inclusions.


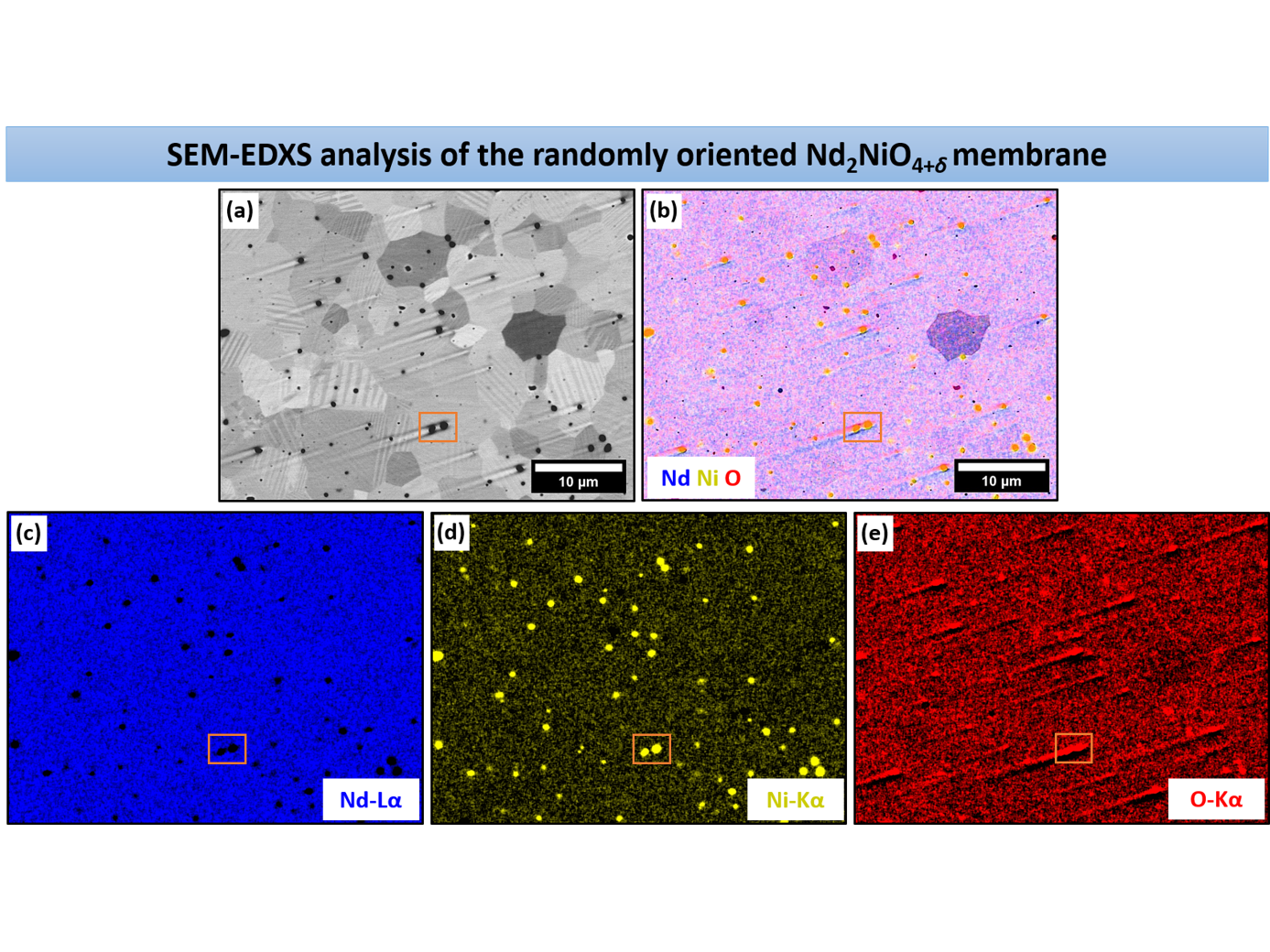


**Figure S7.** a) SEM micrograph of the membrane cross-section with random grain orientation, using backscattered-electron channeling contrast as a basis for EDXS examination. b) EDXS color mixing map resulting from the elemental maps of c) Nd (blue), d) Ni (yellow), and O (red). The NiO inclusions are indicated by regions marked in orange.


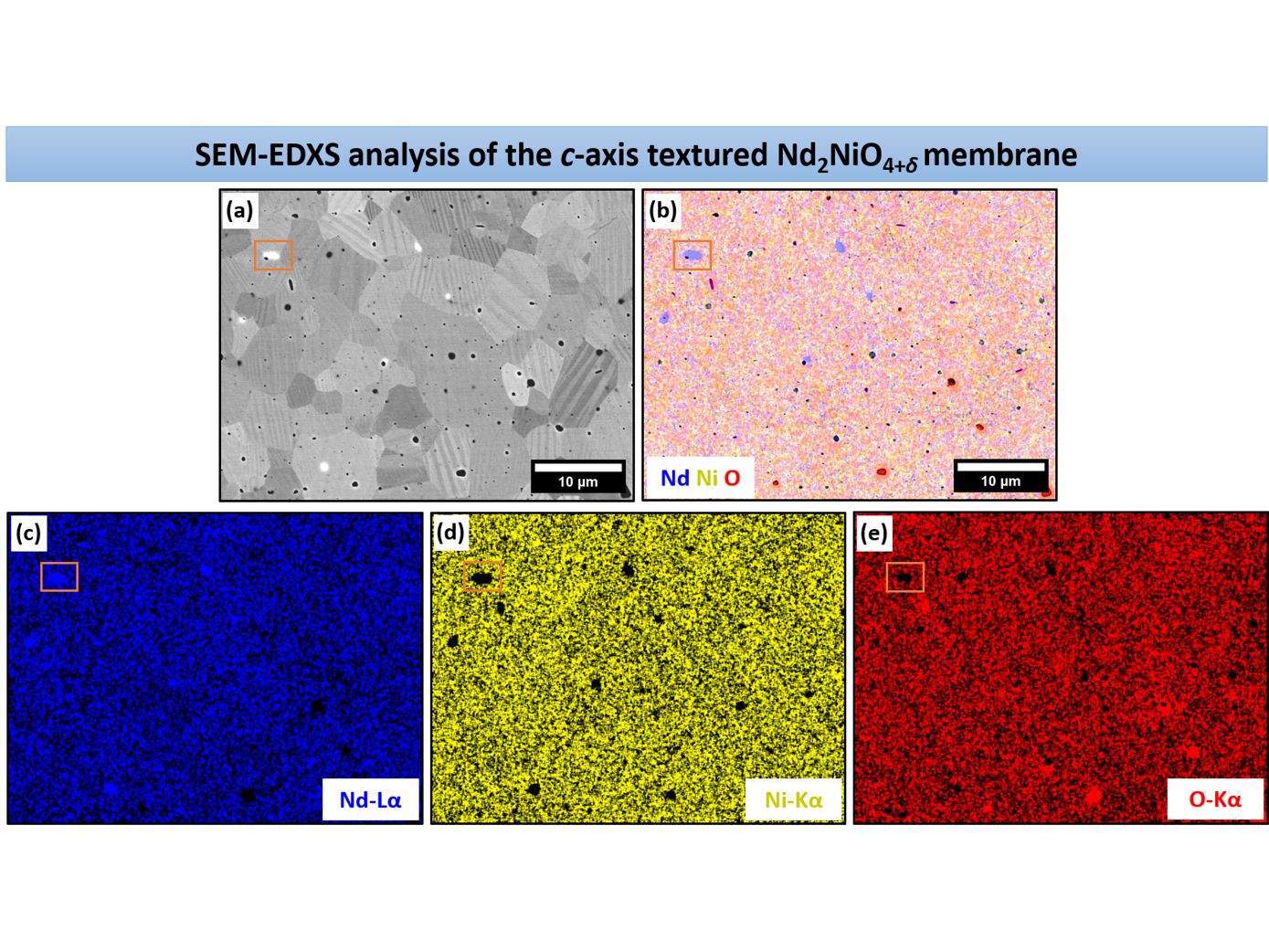


**Figure S8.** a) Backscattered-electron SEM micrographs of the cross-section of the c-axis textured NNO bulk ceramic, serving as the EDXS analysis area. b) EDXS color mixing map generated from the elemental maps of c) Nd (blue), d) Ni (yellow), and O (red). The orange rectangles depict areas containing elemental Nd.


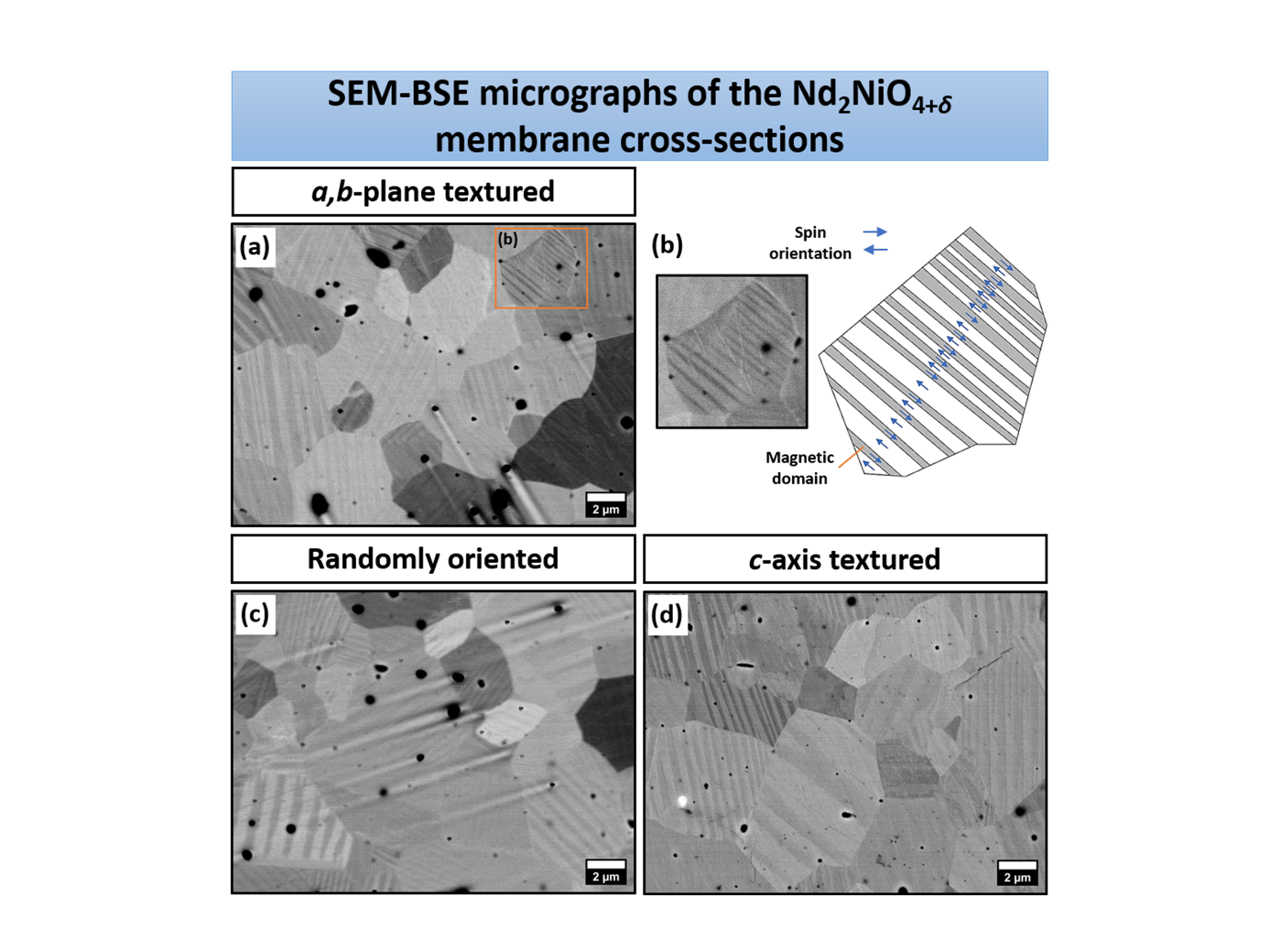


**Figure S9.** a) SEM-BSE micrographs of the vibratory-polished cross-section of the *a*,*b*-plane textured NNO ceramic. b) Selected area in a) with a representation of the grain from the SEM image cutout, indicating the magnetic domains and possible spin orientation due to the antiferromagnetic behavior of NNO in a magnetic field.^[6–8]^ SEM-BSE images displaying the cross-sections of c) the randomly oriented and d) the *c*-axis textured NNO membranes.

**Table S2.** Elemental composition of the ceramic samples based on the EDXS investigations.

| NNO membrane | wt [%] | | | at [%] | | | Stochiometric ratio^a)^ | | |
| --- | --- | --- | --- | --- | --- | --- | --- | --- | --- |
|  | Nd | Ni | O | Nd | Ni | O | Nd | Ni | O |
| *a*,*b*-plane textured | 68.4 ± 4.77 | 15.0 ± 0.46 | 16.3 ± 0.39 | 27.13 ± 1.89 | 15.05 ± 0.47 | 57.81 ± 1.39 | 2.00 ± 0.14 | 1.11 ± 0.03 | 4.26 ± 0.10 |
| randomly oriented | 68.0 ± 4.57 | 15.8 ± 0.50 | 15.9 ± 0.38 | 27.06 ± 1.82 | 15.78 ± 0.49 | 57.16 ± 1.36 | 2.00 ± 0.13 | 1.16 ± 0.04 | 4.22 ± 0.10 |
| *c*-axis textured | 68.8 ± 4.82 | 15.1± 0.43 | 16.1 ±  0.38 | 27.38 ± 1.92 | 14.82 ± 0.42 | 57.80 ± 1.38 | 2.00 ± 0.14 | 1.08 ± 0.03 | 4.22 ± 0.10 |

^a)^ Normalized by the atomic percent of Nd

**Calculation of the NiO precipitation fractions**

To estimate the mass fraction of NiO (wt%_NiO_) in the ceramic membranes, the theoretical mass fraction of Ni (wt%_Ni,theo_) in NNO is required and can be determined using **Equation S2**.

$\text{wt\%}_{\text{Ni,theo}}\text{=}\frac{\text{M}\text{(Ni)}}{\text{M}\text{(}\text{Nd}_{\text{2}}\text{Ni}\text{O}_{\text{4}}\text{)}}\text{=}\frac{\text{58.69} \text{g mol}^{\text{-1}}}{\text{411.17} \text{g mol}^{\text{-1}}}\text{×100}\text{ }\text{=}\text{ }\text{14.}\text{3}\text{ \%}$ (S2)

where *M*(Ni) and *M*(Nd_2_NiO_4_) are the mol masses of Ni and Nd_2_NiO_4_, respectively. The difference between the experimental mass fraction of Ni$(\text{wt\%}_{\text{Ni,exp}}$) from **Table S2** and the theoretical value ($\text{wt\%}_{\text{Ni,theo}}$) indicates the excess nickel (wt%_Ni,excess_) present in the NNO samples, which can be interpreted as NiO. Using **Equation S3,** wt%_NiO_ can be calculated as follows:

$\text{wt\%}_{\text{NiO}}\text{=}\frac{\text{wt\%}_{\text{Ni,excess}}}{\text{M}\text{(Ni)}}\text{× }\text{M}\text{(}\text{NiO}\text{)}$ (S3)

Here *M*(NiO) represents the mol mass of NiO with a value of 74.69 g mol^−1^. **Table S3** summarizes all the data employed to determine wt% _NiO_ in all three NNO membranes.

**Table S3.** NiO precipitation content calculated in NNO ceramic membranes.

| NNO  membrane | $\text{wt\%}_{\text{Ni,exp}}$  [%] | wt%_Ni,theo_  [%] | wt%_Ni,excess_  [%] | $\text{wt\%}_{\text{NiO}}$  [%] |
| --- | --- | --- | --- | --- |
| *a*,*b*-plane textured | 15.0 | 14.3 | 0.7 | 0.89 |
| randomly oriented | 15.8 |  | 1.5 | 1.90 |
| *c*-axis textured | 15.1 |  | 0.8 | 1.01 |

**Table S4**. Estimated values for oxygen bulk diffusion *D**, oxygen surface exchange *k**, and resulting characteristic membrane thickness *L*_c_ at 1173 K using data from the literature and **Equation S4**.^[9]^

| NNO sample | *D** [cm^2^ s^−1^] | *k** [cm s^−1^] | *L*_c_ [mm] | Measurement technique | Ref. |
| --- | --- | --- | --- | --- | --- |
| polycrystalline bulk ceramic | 2.6·10^−7^ | 1.6·10^−6^ | 1.6 | IEDP/SIMS^a)^ | [10] |
| polycrystalline bulk ceramic | 1.1·10^−4^ | 4.6·10^−2^ | 0.2 | ECR^b)^ | [11] |

^a)^IEDP/SIMS: Isotope exchange depth profiling combined with secondary ion mass-spectrometry, ^b)^ECR: Electrical conductivity relaxation

$\text{L}_{\text{c}}\text{ =}\text{ }\frac{\text{D}\text{*}}{\text{k}\text{*}}$ (S4)

**Table S5**. Calculated values for oxygen bulk diffusion *D**, oxygen surface exchange *k**, and characteristic membrane thickness *L*_c_ at 1073 K using literature data and **Equation S4**.

| NNO sample | *D** [cm^2^ s^−1^] | *k** [cm s^−1^] | *L*_c_ [mm] | Measurement technique | Ref. |
| --- | --- | --- | --- | --- | --- |
| polycrystalline bulk ceramic | 9.8·10^−8^ | 7.7·10^−7^ | 1.3 | IEDP/SIMS^a)^ | [10] |
| polycrystalline bulk ceramic | 5.6·10^−5^ | 4.3·10^−3^ | 1.3 | ECR^b)^ | [11] |
| polycrystalline bulk ceramic | 2.3·10^−7^ | 2.1·10^−7^ | 10.9 | IE/GPE^c)^ | [12] |
| polycrystalline bulk ceramic | 1.5·10^−7^ | 8.0·10^−7^ | 1.9 | IEDP/SIMS^a)^ | [13] |

^a)^IEDP/SIMS: Isotope exchange depth profiling combined with secondary ion mass-spectrometry, ^b)^ECR: Electrical conductivity relaxation, ^c)^IE/GPE: Isotope exchange with gas phase equilibration

**Table S6**. Values for oxygen bulk diffusion *D**, oxygen surface exchange *k**, and characteristic membrane thickness *L*_c_ at 973 K, obtained using literature data and **Equation S4**.

| NNO sample | *D** [cm^2^ s^−1^] | *k** [cm s^−1^] | *L*_c_ [mm] | Measurement technique | Ref. |
| --- | --- | --- | --- | --- | --- |
| polycrystalline bulk ceramic | 2.8·10^−8^ | 3.2·10^−7^ | 0.9 | IEDP/SIMS^a)^ | [10] |
| polycrystalline bulk ceramic | 9.3·10^−6^ | 3.9·10^−4^ | 2.4 | ECR^b)^ | [11] |
| polycrystalline bulk ceramic | 4.6·10^−8^ | 5.9·10^−8^ | 7.8 | IE/GPE^c)^ | [12] |
| polycrystalline bulk ceramic | 5.0·10^−8^ | 3.0·10^−7^ | 1.7 | IEDP/SIMS^a)^ | [13] |
| *a*,*b*-plane oriented single crystal | 2.3·10^−7^ | 1.2·10^−6^ | 1.92 | IEDP/SIMS^a)^ | [14] |
| *c-*axis oriented single crystal | 3.0·10^−10^ | 3.9·10^−7^ | 0.01 | IEDP/SIMS^a)^ | [14] |

^a)^IEDP/SIMS: Isotope exchange depth profiling combined with secondary ion mass-spectrometry, ^b)^ECR: Electrical conductivity relaxation, ^c)^IE/GPE: Isotope exchange with gas phase equilibration

**Table S7**. Reported activation energy values for the oxygen bulk diffusion *E*_a_ (*D**) and surface exchange *E*_a_ (*k**) in various NNO-based materials from the literature.

| NNO sample | Measurement method | *E*_a_ (*D**)  [eV] | *E*_a_ (*k**)  [eV] | Temperature range  [K] | Ref. |
| --- | --- | --- | --- | --- | --- |
| *a*,*b*-plane oriented single crystal | IEDP/SIMS^a)^ | 1.40 | 1.10 | 723−973 | [14] |
| *c-*axis oriented single crystal | IEDP/SIMS^a)^ | 1.30 | 1.80 | 723−973 | [14] |
| polycrystalline ceramic | IEDP/SIMS^a)^ | 1.06 | 0.77 | 773−1073 | [10] |
| polycrystalline ceramic | IE/GPE^b)^ | 1.45 | − | 873−1123 | [12] |
| polycrystalline ceramic | ECR^c)^ | 0.88 | − | 973−1173 | [11] |
| polycrystalline ceramic | ECR^c)^ | − | 1.10 | 873−1173 | [15] |
| polycrystalline ceramic | ECR^c)^ | 0.90 | 1.43 | 773−973 | [16] |

^a)^IEDP/SIMS: Isotope exchange depth profiling combined with secondary ion mass-spectrometry, ^b)^IE/GPE: Isotope exchange with gas phase equilibration, ^c)^ECR: Electrical conductivity relaxation

**Table S8**. Binding energies of various oxygen species in the O 1s XPS spectra of fresh and spent NNO ceramic surfaces.

| NNO membrane | $O_{\mathrm{latt}}$[eV] | $O^{2-}$[eV] | $O^{-}$[eV] | $O_{2}^{-}$ [eV] | $\mathrm{OH}^{-}$[eV] |
| --- | --- | --- | --- | --- | --- |
| *a*,*b*-plane textured (fresh) | 528.64 | 529.04 | 530.74 | 531.40 | 532.74 |
| *a*,*b*-plane textured (spent) | 529.85 | 530.25 | 531.95 | 532.61 | 533.95 |
| *c*-axis textured (fresh) | 529.29 | 529.69 | 531.39 | 532.05 | 533.39 |
| *c*-axis textured (spent) | 528.61 | 529.03 | 530.52 | 531.49 | 532.74 |

**Table S9**. Fitted XPS area results for various oxygen species in the O 1s XPS spectra of fresh and spent NNO ceramic surfaces.

| NNO membrane | $O_{\mathrm{latt}}$ | $O^{2-}$ | $O^{-}$ | $O_{2}^{-}$ | $\mathrm{OH}^{-}$ | $O_{\mathrm{ads}} / O_{\mathrm{latt}}$ |
| --- | --- | --- | --- | --- | --- | --- |
| *a*,*b*-plane textured (fresh) | 16803 | 8574 | 5596 | 2750 | 61 | 1.01 |
| *a*,*b*-plane textured (spent) | 636 | 2873 | 511 | 2007 | 32 | 8.53 |
| *c*-axis textured (fresh) | 8909 | 3817 | 941 | 745 | 25 | 0.62 |
| *c*-axis textured (spent) | 22512 | 11539 | 7220 | 4378 | 141 | 1.03 |


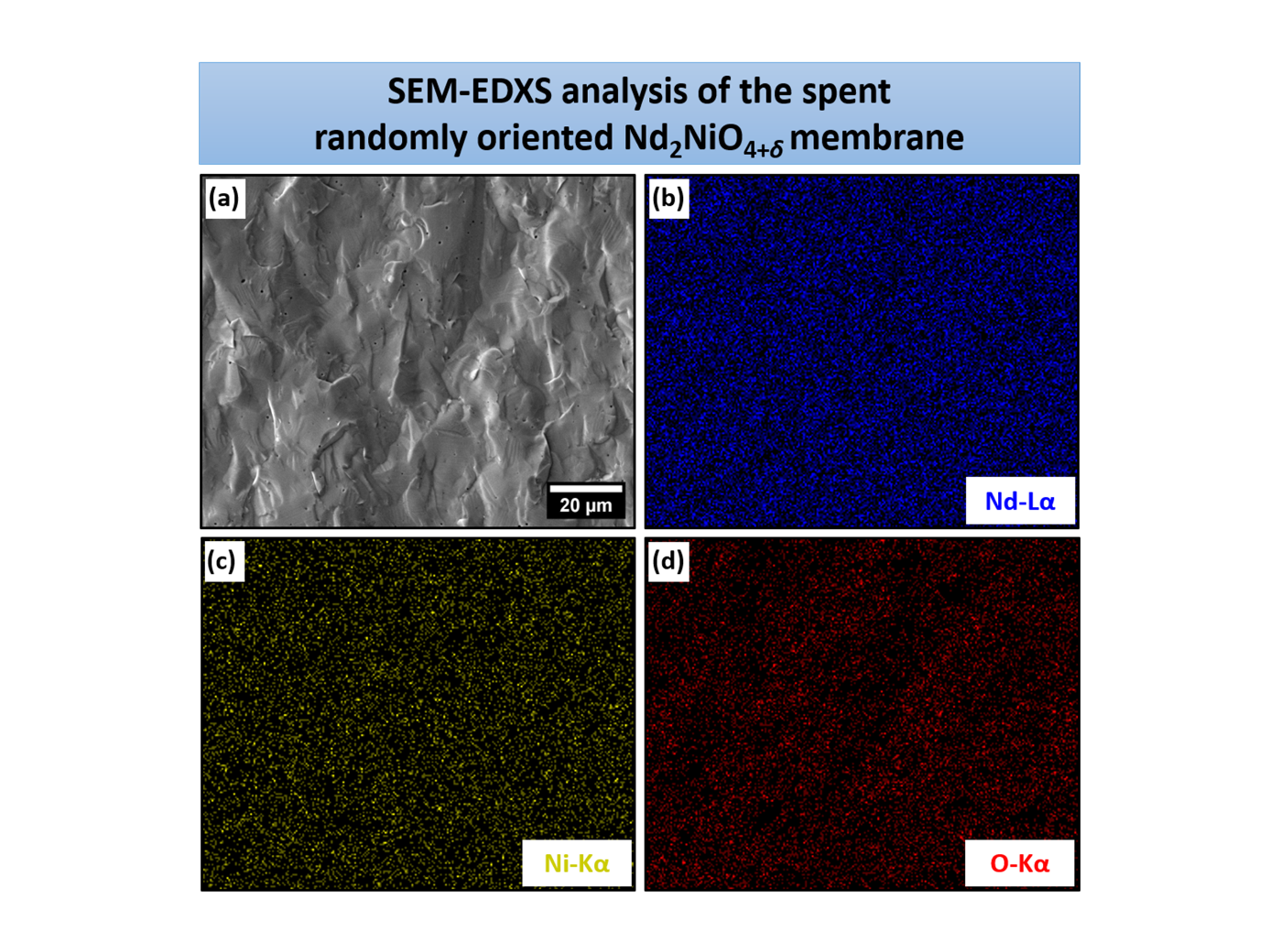


**Figure S10.** a) SEM micrograph displaying the fractured cross-section of the spent polycrystalline NNO membrane, which serves as the analysis area for EDXS. (b-d) Corresponding EDXS element maps for Nd (blue), Ni (yellow), and O (red).


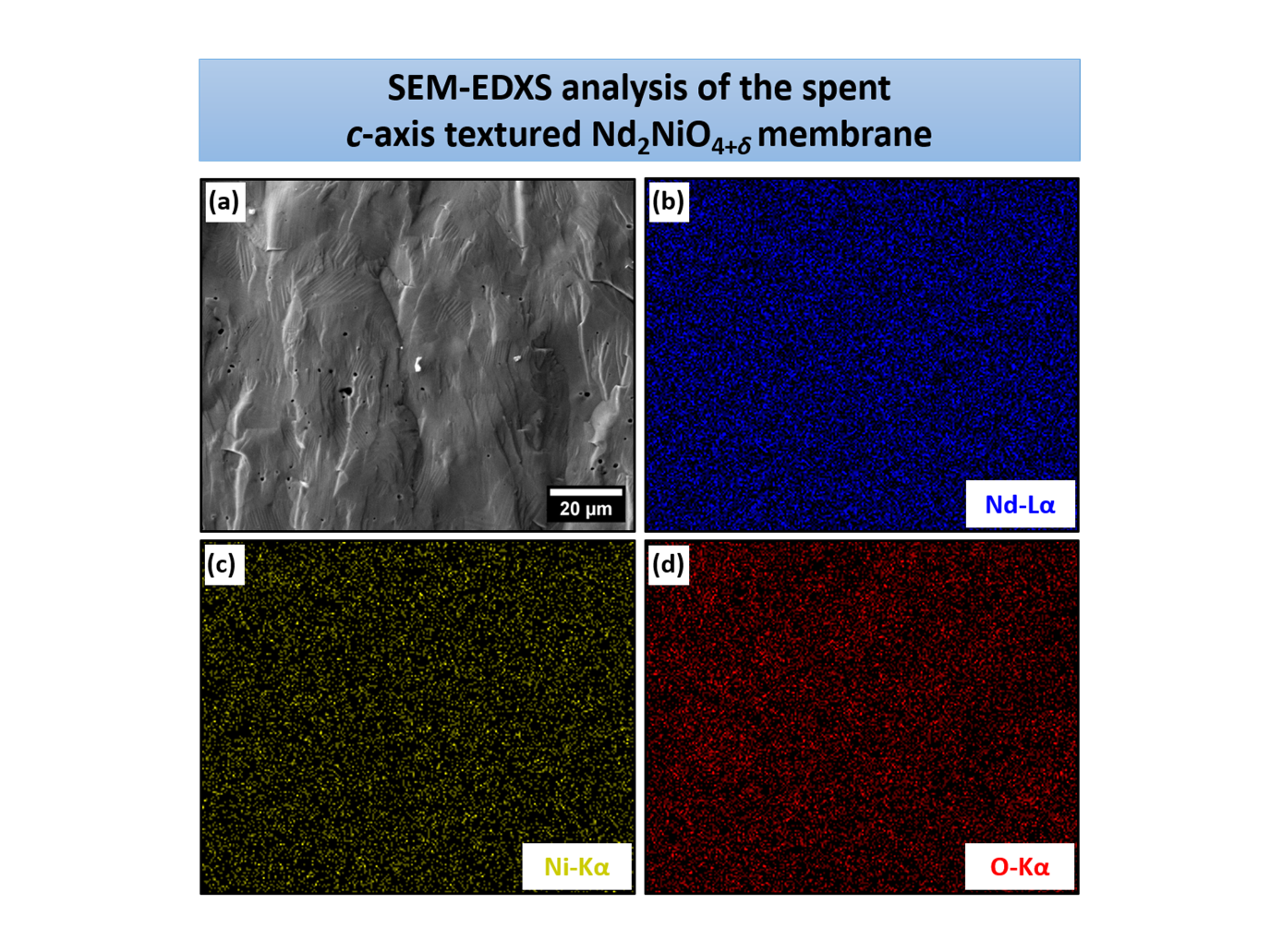


**Figure S11.** a) SEM micrograph as basis for the EDXS analysis, presenting the fractured cross-section of the spent *c*-axis textured NNO ceramic. (b-d) Resulting EDXS element maps for Nd (blue), Ni (yellow), and O (red).

**Table S10**. Overview of oxygen permeation fluxes in disk-shaped bulk ceramic membranes under air/He or air/CO₂ gradients at various temperatures recently described in the literature.

| Bulk membrane | Structure  type | Membrane thickness  [mm] | Oxygen flux (He sweep) [mL min^−1^ cm^−2^] | Oxygen flux (CO_2_ sweep)  [mL min^−1^ cm^−2^] | Stability time (CO_2_ sweep) [h] | Ref. |
| --- | --- | --- | --- | --- | --- | --- |
| *a*,*b*-plane textured Nd_2_NiO_4+δ_ | K_2_NiF_4_ | 1.00 | 1.02 (1223 K) | 0.74 (1223 K) | 140 | this work |
| Nd_2_NiO_4+δ_ | K_2_NiF_4_ | 1.00 | 0.47 (1223 K) | − | − | this work |
| Nd_2_NiO_4+δ_ | K_2_NiF_4_ | 1.00 | 0.53 (1223 K) | − | − | [17] |
| Nd_2_NiO_4+δ_ | K_2_NiF_4_ | 1.00 | 0.04 (1173 K) | − | − | [18] |
| La_2_NiO_4+δ_ | K_2_NiF_4_ | 1.00 | 0.47 (1223 K) | 0.22 (1173 K) | 120 | [19] |
| (La_0.9_Ca_0.1_)_2_(Ni_0.75_Cu_0.25_)O_4+_*_δ_* | K_2_NiF_4_ | 0.65 | 0.65 (1173 K) | 0.63 | − | [20] |
| (Pr_0.9_La_0.1_)_1.9_Ni_0.74_Cu_0.21_Ga_0.5_O_4+_*_δ_* modified with catalytic La_0.6_Sr_0.4_CoO_3_ layer | K_2_NiF_4_ | 0.60 | 0.90 (1173 K) | − | − | [21] |
| (Pr_0.9_La_0.1_)_2_(Ni_0.74_Cu_0.21_Ga_0.05_)O_4+_*_δ_* | K_2_NiF_4_ | 1.00 | 0.30 (1223 K) | 0.27 (1223 K) | − | [22] |
| (Nd_0.9_La_0.1_)_2_(Ni_0.74_Cu_0.21_Al_0.05_)O_4+_*_δ_* | K_2_NiF_4_ | 0.60 | 0.50 (1223 K) | 0.39 (1248 K) | 250 | [23] |
| Pr_0.5_Sr_0.5_Cu_0.2_Fe_0.8_O_3−_*_δ_* | Perovskite | 1.40 | 0.40 (1173 K) | 0.16 (1173 K) | 100 | [24] |
| Ba_0.5_Sr_0.5_Fe_0.8_Zn_0.2_O_3−_*_δ_* | Perovskite | 1.15 | 1.08 (1223 K) | − | − | [25] |
| Ba_0.5_Sr_0.5_Co_0.8_Fe_0.2_O_3−_*_δ_* | Perovskite | 0.50 | 2.30 (1223 K) | 0.00 (1223 K) | 1 | [26] |
| Ce_0.9_Gd_0.1_O_2−_*_δ_* − Ba_0.5_Sr_0.5_Co_0.8_Fe_0.2_O_3−_*_δ_* | Dual-phase | 0.50 | 1.30 (1223 K) | 0.67 (1223 K) | 250 | [26] |
| 60 wt% Ce_0.9_Nd_0.1_O_2−_*_δ_* – 40 wt% Nd_0.6_Sr_0.4_CoO_3−_*_δ_* | Dual-phase | 0.60 | 0.65 (1223 K) | 0.55 (1223 K) | 150 | [27] |
| 60 wt% Ce_0.9_Pr_0.1_O_2−_*_δ_* − 40 wt% Pr_0.6_Sr_0.4_Fe_0.5_Co_0.5_O_3−_*_δ_* | Dual-phase | 0.60 | 0.84 (1223 K) | 0.70 (1223 K) | 400 | [28] |

**References**

[1] T. Uchikoshi, T. S. Suzuki, F. Tang, H. Okuyama, Y. Sakka, *Ceram. Int.* **2004**, *30*, 1975.

[2] T. Kimura, T. Uemura, T. Kimura, S. Takagi, H. Inoue, *Macromol. Symp.* **2006**, *242*, 120.

[3] T. S. Suzuki, Y. Sakka, K. Kitazawa, *Adv. Eng. Mater.* **2001**, *3*, 490.

[4] S. R. Maity, M. Ceretti, L. Keller, J. Schefer, T. Shang, E. Pomjakushina, M. Meven, D. Sheptyakov, A. Cervellino, W. Paulus, *Phys. Rev. Mater.* **2019**, *3*, 083604.

[5] S. Koizumi, T. S. Suzuki, Y. Sakka, K. Yabe, T. Hiraga, *Phys. Chem. Miner.* **2016**, *43*, 689.

[6] A. Romano, E. S. Şuhubi, *Int. J. Eng. Sci.* **1992**, *30*, 1715.

[7] J. Taurines, M. Olive, R. Desmorat, O. Hubert, B. Kolev, *J. Magn. Magn. Mater.* **2020**, *546*, 167885.

[8] S. F. Weber, A. Urru, S. Bhowal, C. Ederer, N. A. Spaldin, *Phys. Rev. X* **2024**, *14*, 21033.

[9] C. H. Chen, H. J. M. Bouwmeester, R. H. E. Van Doom, H. Kruidhof, A. J. Burggraaf, *Solid State Ionics* **1997**, *98*, 7.

[10] E. Boehm, J. M. Bassat, P. Dordor, F. Mauvy, J. C. Grenier, P. Stevens, *Solid State Ionics* **2005**, *176*, 2717.

[11] J. Song, D. Ning, B. Boukamp, J. M. Bassat, H. J. M. Bouwmeester, *J. Mater. Chem. A* **2020**, *8*, 22206.

[12] E. Y. Pikalova, V. A. Sadykov, E. A. Filonova, N. F. Eremeev, E. M. Sadovskaya, S. M. Pikalov, N. M. Bogdanovich, J. G. Lyagaeva, A. A. Kolchugin, L. B. Vedmid, A. V Ishchenko, V. B. Goncharov, *Solid State Ionics* **2019**, *335*, 53.

[13] F. Mauvy, J. M. Bassat, E. Boehm, J. P. Manaud, P. Dordor, J. C. Grenier, *Solid State Ionics* **2003**, *158*, 17.

[14] J. M. Bassat, M. Burriel, O. Wahyudi, R. Castaing, M. Ceretti, P. Veber, I. Weill, A. Villesuzanne, J. Grenier, W. Paulus, J. A. Kilner, *J. Phys. Chem. C* **2013**, *117*, 26466.

[15] T. Ina, Y. Orikasa, T. Masese, T. Nakao, A. Mineshige, K. Amezawa, H. Tanida, T. Uruga, Y. Uchimoto, *Electrochemistry* **2014**, *82*, 875.

[16] K. Yakal-Kremski, L. V. Mogni, A. Montenegro-Hernández, A. Caneiro, S. A. Barnett, *J. Electrochem. Soc.* **2014**, *161*, F1366.

[17] Z. Zhao, G. Chen, G. Escobar Cano, P. A. Kißling, O. Stölting, B. Breidenstein, S. Polarz, N. C. Bigall, A. Weidenkaff, A. Feldhoff, *Angew. Chemie - Int. Ed.* **2024**, *63*, e202312473.

[18] P. M. Geffroy, M. Reichmann, T. Chartier, J. M. Bassat, J. C. Grenier, *J. Memb. Sci.* **2014**, *451*, 234.

[19] T. Klande, K. Efimov, S. Cusenza, K. D. Becker, A. Feldhoff, *J. Solid State Chem.* **2011**, *184*, 3310.

[20] G. Chen, M. Widenmeyer, B. Tang, L. Kaeswurm, L. Wang, A. Feldhoff, A. Weidenkaff, *Front. Chem. Sci. Eng.* **2020**, *14*, 405.

[21] J. Xue, G. Weng, L. Chen, Y. Suo, Y. Wei, A. Feldhoff, H. Wang, *J. Memb. Sci.* **2019**, *573*, 588.

[22] J. Tang, Y. Wei, L. Zhou, Z. Li, H. Wang, *AIChE J.* **2011**, *58*, 2473.

[23] Y. Chen, Q. Liao, Y. Wei, Z. Li, H. Wang, *Ind. Eng. Chem. Res.* **2013**, *52*, 8571.

[24] Z. Wang, W. Liu, Y. Wu, W. Sun, C. Wang, *J. Memb. Sci.* **2019**, *573*, 504.

[25] J. Martynczuk, M. Arnold, A. Feldhoff, *J. Memb. Sci.* **2008**, *322*, 375.

[26] J. Xue, Q. Liao, Y. Wei, Z. Li, H. Wang, *J. Memb. Sci.* **2013**, *443*, 124.

[27] Y. He, L. Shi, F. Wu, W. Xie, S. Wang, D. Yan, P. Liu, M. R. Li, J. Caro, H. Luo, *J. Mater. Chem. A* **2017**, *6*, 84.

[28] F. Liang, H. Luo, K. Partovi, O. Ravkina, Z. Cao, Y. Liu, J. Caro, *Chem. Commun.* **2014**, *50*, 2451.
